# Identification of key genes by integrating DNA methylation and next-generation transcriptome sequencing for esophageal squamous cell carcinoma

**DOI:** 10.18632/aging.102686

**Published:** 2020-01-21

**Authors:** Yang Chen, Lian-Di Liao, Zhi-Yong Wu, Qian Yang, Jin-Cheng Guo, Jian-Zhong He, Shao-Hong Wang, Xiu-E Xu, Jian-Yi Wu, Feng Pan, De-Chen Lin, Li-Yan Xu, En-Min Li

**Affiliations:** 1 The Key Laboratory of Molecular Biology for High Cancer Incidence Coastal Chaoshan Area, Shantou University Medical College, Shantou 515041, Guangdong, P.R. China; 2 Department of Biochemistry and Molecular Biology, Shantou University Medical College, Shantou 515041, Guangdong, P.R. China; 3 Institute of Oncologic Pathology, Shantou University Medical College, Shantou 515041, Guangdong, P.R. China; 4 Departments of Oncology Surgery, Shantou Central Hospital, Affiliated Shantou Hospital of Sun Yat-Sen University, Shantou 515041, Guangdong, P.R. China; 5 Departments of Pathology, Shantou Central Hospital, Affiliated Shantou Hospital of Sun Yat-Sen University, Shantou 515041, Guangdong, P.R. China; 6 Department of Medicine, Cedars-Sinai Medical Center, Los Angeles, CA 90048, USA

**Keywords:** esophageal squamous cell carcinoma, DNA methylation, next-generation sequencing, histone modification, survival analysis

## Abstract

Aberrant DNA methylation leads to abnormal gene expression, making it a significant regulator in the progression of cancer and leading to the requirement for integration of gene expression with DNA methylation. Here, we identified 120 genes demonstrating an inverse correlation between DNA methylation and mRNA expression in esophageal squamous cell carcinoma (ESCC). Sixteen key genes, such as SIX4, CRABP2, and EHD3, were obtained by filtering 10 datasets and verified in paired ESCC samples by qRT-PCR. 5-Aza-dC as a DNA methyltransferase (DNMT) inhibitor could recover their expression and inhibit clonal growth of cancer cells in seven ESCC cell lines. Furthermore, 11 of the 16 genes were correlated with OS (overall survival) and DFS (disease-free survival) in 125 ESCC patients. ChIP-Seq data and WGBS data showed that DNA methylation and H3K27ac histone modification of these key genes displayed inverse trends, suggesting that there was collaboration between DNA methylation and histone modification in ESCC. Our findings illustrate that the integrated multi-omics data (transcriptome and epigenomics) can accurately obtain potential prognostic biomarkers, which may provide important insight for the effective treatment of cancers.

## INTRODUCTION

Esophageal cancer is globally one of the most prevalent cancers which is diagnosed more than 500,000 new cases yearly [1]. Esophageal adenocarcinoma (EAC) and esophageal squamous cell carcinoma (ESCC) are the two main clinical subtypes of esophageal cancer, with more than 80% of esophageal cancers being ESCC [2]. In Asia, ESCC has higher morbidity and mortality compared with western countries [3]. More than half of patients with these tumors have distal metastases at the time of diagnosis, and only 10%–20% of sufferers survive for 5 years [1]. Therefore, there is an urgent need to provide effective targets for therapy or early detection of ESCC.

Epigenetic alterations have been suggested as underlying mechanisms of cancer progression [4]. DNA methylation is one of the major epigenetic mechanisms involved in cancer [5], with global DNA hypomethylation accompanied by hypermethylation of tumor suppressor genes being recognized as an epigenetic hallmark of cancer [6]. Altered DNA methylation patterns can change gene expression by silencing or activating genes, so numerous studies aimed at revealing the pathogenesis of tumors have focused on epigenetics, such as circulating tumor DNA (ctDNA) analysis [7–9] and whole-genome bisulfite sequencing (WGBS) [10–12]. In 1999, the feasibility of detecting tumor-associated abnormal ctDNA methylation was initially described [13]. Since then, more and more studies have been conducted to characterize the potential use of ctDNA methylation for early diagnosis and prognosis [14]. Some methylation-associated drugs, such as decitabine, an effective drug for myelodysplastic syndrome (MDS) and acute myeloid leukemia (AML), have been used for the clinical therapy of AML [15]. On the other hand, next-generation transcriptome sequencing (RNA-Seq) provides a method for tracing transcriptional aberrations in diseases [16, 17]. In addition, posttranslational modifications of histones also regulate gene activity in tumors [18, 19]. Nevertheless, there is a little research on integrating DNA methylation and gene expression or histone modification in ESCC.

Here, we systematically define DNA methylation status and mRNA expression level and demonstrate the correlation between DNA methylation and gene expression by using DNA methylation array and RNA-seq data. Furthermore, we perform a comprehensive analysis to determine whether some identified genes could serve as potential prognostic biomarkers and could be applied to the treatment of ESCC.

## RESULTS

### Identification of differentially-methylated genes

**Figure 1:**
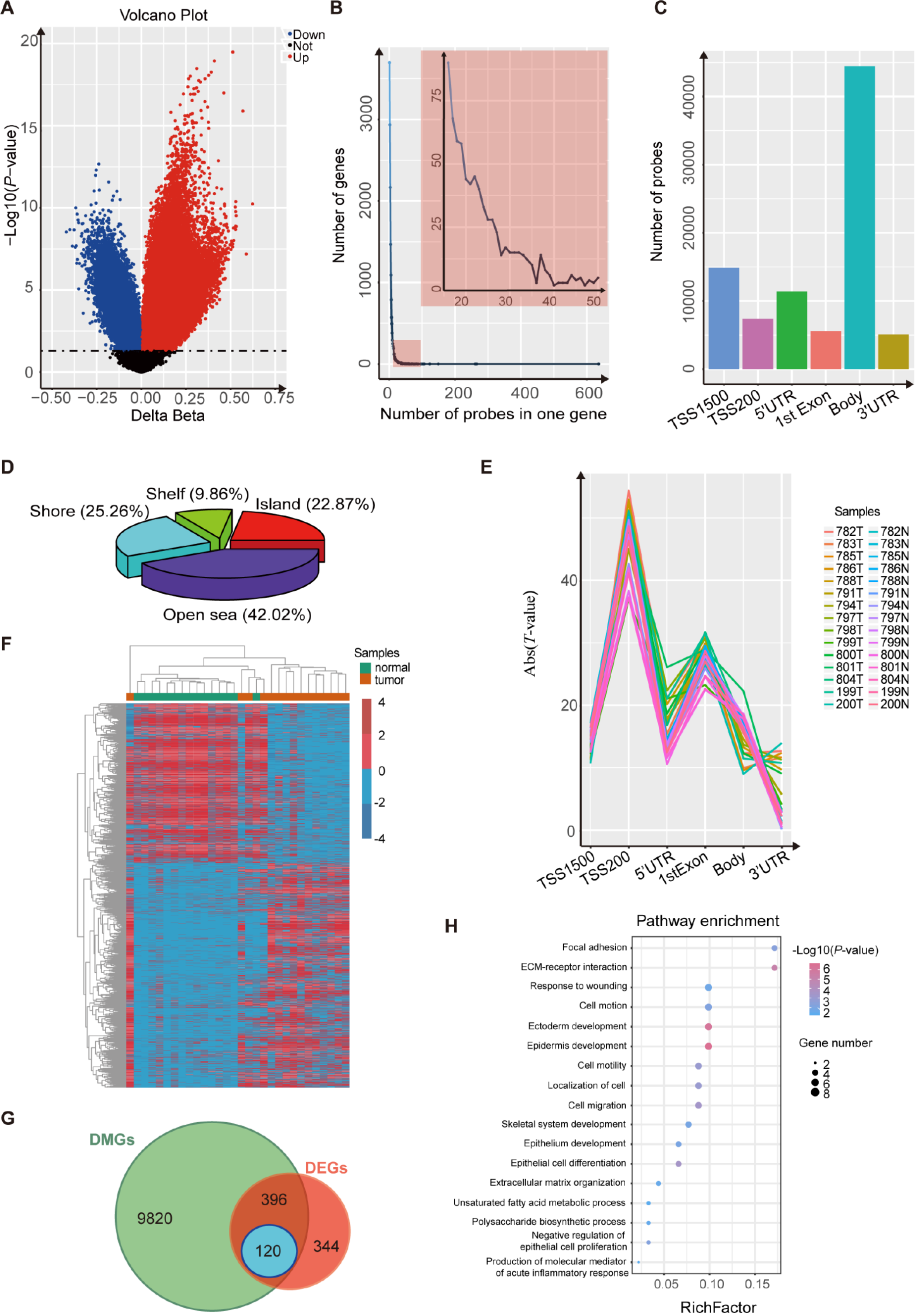
**Analysis using the Illumina 450K bead array and RNA-Seq data for 15 paired ESCC samples.** (**A**) Volcano plot representing the probes for differentially-methylated genes. The probes for hypermethylated genes are shown in red while probes for hypomethylated genes are shown in blue (*P*< 0.05). (**B**) Frequency line graph shows the coverage rate of differentially-methylated regions (DMPs) in one gene. The results show that most genes have more than twenty DMPs. (**C**) Distribution of differentially-methylated sites in six gene regions (TSS1500, TSS200, 5’ UTR, 1^st^ exon, gene body and 3’ UTR). (**D**) Proportions of differentially-methylated regions from genes with associated CpG islands (CGI). (**E**) Plot of the regression t-statistics between log-normalized RNA-Seq RPKM values and corresponding average DNA methylation *β* values for probes, stratified according to six genetic regions. The number of curves equals the number of samples. (**F**) Heatmap shows the differentially-methylated genes in 15 paired ESCC samples. (**G**) Venn plot shows the overlap between differentially-methylated genes and differentially-expressed genes in 15 paired ESCC samples. (**H**) KEGG and GO analysis of 120 candidate genes that are both differentially methylated and differentially expressed.

We performed an Illumina Infinium HumanMethylation450 BeadChip assay to compare esophageal DNA patterns in fifteen ESCC patients’ tumor tissues and paired normal tissues. After the chip was pretreated, the methylation data of the 15 pairs of esophageal squamous cell carcinoma samples was analyzed using the *minfi* package. We detected 80,557 CpG sites, related to 15,882 different genes, that showed significant differences in DNA methylation in ESCC (*p*-value<0.05, Figure 1A and Supplementary Table 1). We also found that most of the genes were covered with 1~15 differentially-methylated probes (DMPs) (Figure 1B). Most of the 80,557 differentially-methylated sites were located in the gene body and TSS1500 (Figure 1C), and more than half of the DMPs were located around the C_P_G islands (Figure 1D). Because several probes can map to a given gene when using the Illumina Infinium 450k bead array, we wanted to evaluate which probes were most relevant to the state of gene expression. To determine this, we averaged the DNA methylation β value of probes mapping to the same gene region (TSS1500, TSS200, 5’UTR, 1^st^ exon, gene body and 3’UTR). Regression analysis revealed that the strongest associations between gene expression and DNA methylation were for TSS200, 1^st^ exon and TSS1500 regions (Figure 1E). Finally, we assigned a unique β value to a given gene by the following scheme: for a gene with probes binding to TSS200, the average β value of such TSS200 probes was used. For a gene with no TSS200 probes but with probes binding to the 1^st^ exon, we used the average over 1^st^ exon probes. For a gene with no TSS200- or 1^st^ exon-binding probes, the average over the TSS1500 probes was used [20]. After these analyses, we obtained 10,336 differentially-methylated genes (Figure 1F).

### Integration of DNA methylation and mRNA expression data to obtain candidate genes

Expression analysis of genes was performed on 15 paired esophageal samples. We identified 860 differentially-expressed genes between tumors and non-tumor matched samples (Figure 1G; *p*-value<0.05, |log2(FC)|>1). Based on these results, we ultimately obtained 120 candidate genes that were both differentially methylated and expressed, and with the strongest negative associations between gene expression and DNA methylation (Figure 1G; *p*-value<0.05, PCC <-0.5). Then we found that these candidate genes were cancer-associated, by DAVID enrichment analysis, which suggested that abnormal methylation also plays an important role in esophageal squamous cell carcinoma (Figure 1H).

### Use of multiple expression profiles to screen the specific methylated key genes in ESCC

**Figure 2:**
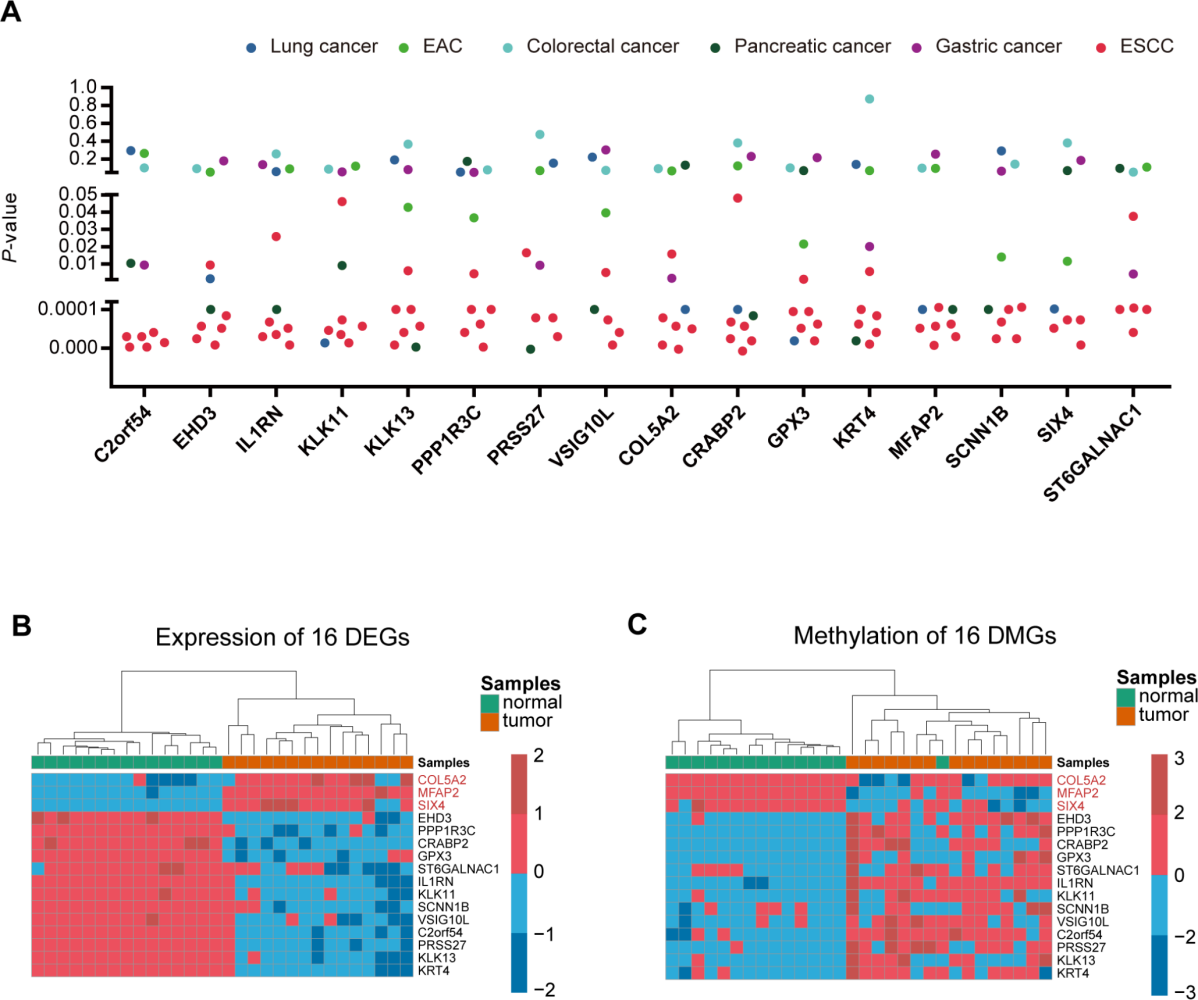
**Identification of ESCC-specific genes.** (**A**), Genes were filtered from ten data sets (5 ESCC data sets and five other cancer data sets) to obtain specific key genes in ESCC. Scatter plot shows the *p-*value of 16 differentially-expressed genes in the ten data sets. (**B**, **C**) Heatmap of methylation and expression of the 16 key genes, respectively.

In order to find the specific genes regulated by DNA methylation only in ESCC, we then downloaded 10 datasets (5 ESCC datasets and 5 other cancer datasets) from the GEO database to filter the 120 candidate genes. The genes that showed differential expression in at least 3 of the 5 ESCC datasets, and differential expression in no more than 2 pan-cancer datasets in 5 other cancers, were selected for key genes. Ultimately, we identified 16 specific genes in ESCC (Figure 2A and Supplementary Table 2). The heatmap of the 16 genes shows the expression level and methylation status in 15 paired ESCC samples (Figure 2B and 2C).

### Key genes regulated by methylation affect the proliferation of ESCC cells

**Figure 3:**
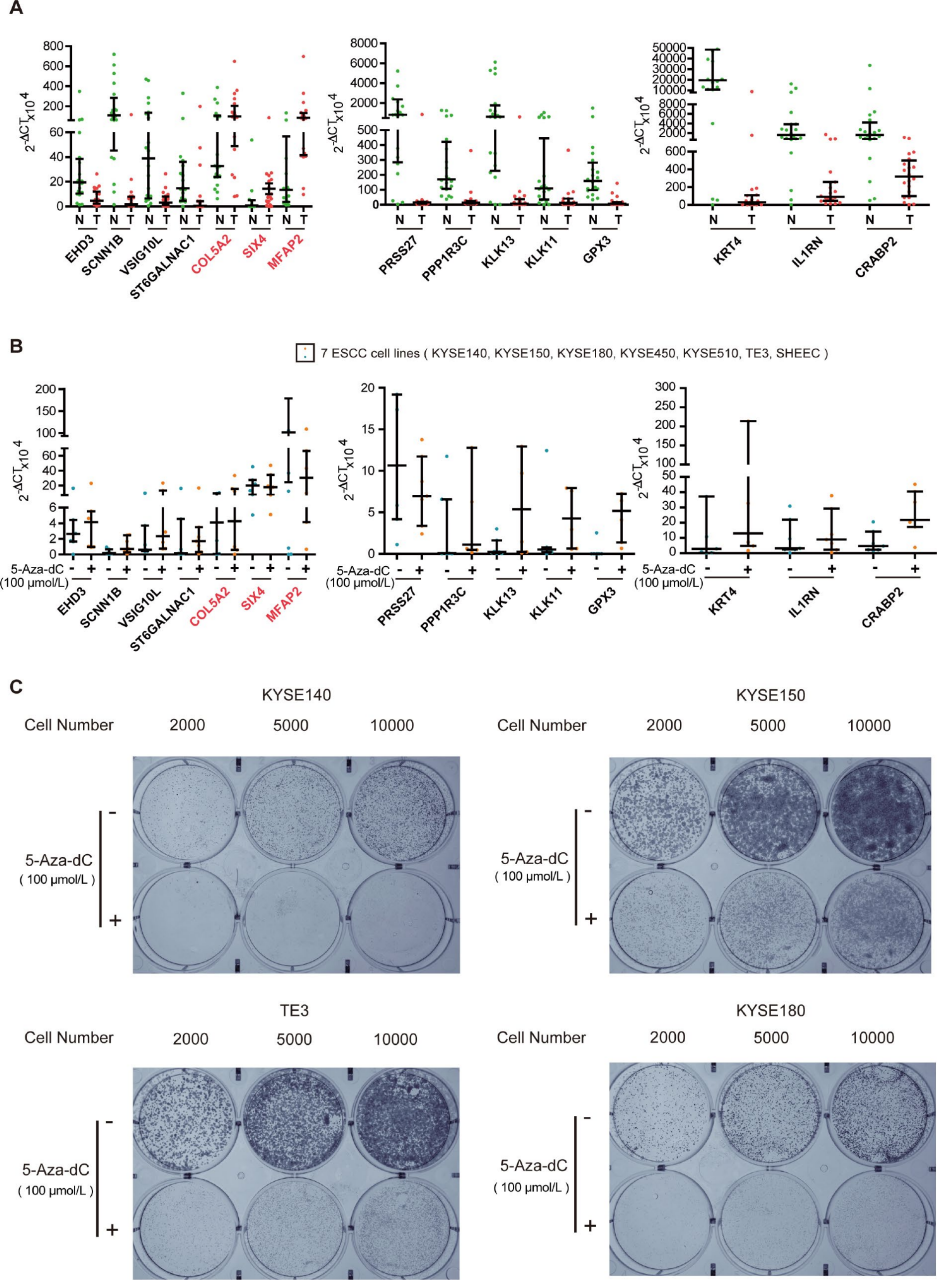
**Experimental verification of key genes in ESCC.** (**A**) qRT-PCR analysis of key genes in tumor (T) and normal (N) tissues of twenty paired ESCC samples. (**B**) Scatter plot of qRT-PCR analyses for key genes in seven ESCC cell lines. Blue spots represent cells treated with DMSO, whereas the orange spots represent cells treated with 5-aza-dC. (**C**) Colony formation assays of ESCC cells after 5-aza-dC treatment. ESCC cells were plated in 6-well plates. After 24 h, the cells were treated with 5-aza-dC. Cultures were maintained for six days, and cells were then stained and photographed. DMSO was used as the control. Colony formation assays illustrate that hypermethylation of key genes plays an important role in cell growth.

To test the biological functions of the 16 genes, we first analyzed expression of the genes, in twenty ESCC tumor tissues and paired normal esophageal epithelial tissues, using qRT-PCR. Expression of most of the 16 genes could be detected in all of the tumors and normal esophageal epithelium tissues, except for C2orf54. As shown in Figure 3A, expression of the hypermethylated genes (VSIG10L, ST6GALNAC1, SCNN1B, PRSS27, PPP1R3C, KRT4, KLK13, KLK11, IL1RN, GPX3, EHD3 and CRABP2) in tumor tissues was lower than that of the corresponding genes in normal esophageal epithelial tissues. The hypomethylated genes (SIX4, MFAP2 and COL5A2) showed the reverse trend. In order to know whether the DNA methylation status of these 15 genes was associated with their expression in ESCC, we further detected their mRNA expression in 5-aza-dC-treated ESCC cell lines. The expression of hypermethylated genes increased in seven ESCC cell lines after 5-aza-dC treatment (Figure 3B). Then, we cultured KYSE140, KYSE150, TE3 and KYSE180 cells with or without 5-aza-dC and examined the ability of cells to form colonies. The efficiency of cell colony formation in the presence of 5-aza-dC was significantly decreased, suggesting that aberrant hypermethylation of these key genes could promote cell colony formation and proliferation of ESCC cells (Figure 3C). All of these results illustrate that methylation-related genes play a vital role in ESCC.

### Evaluation of the prognostic performance of the key genes

**Figure 4:**
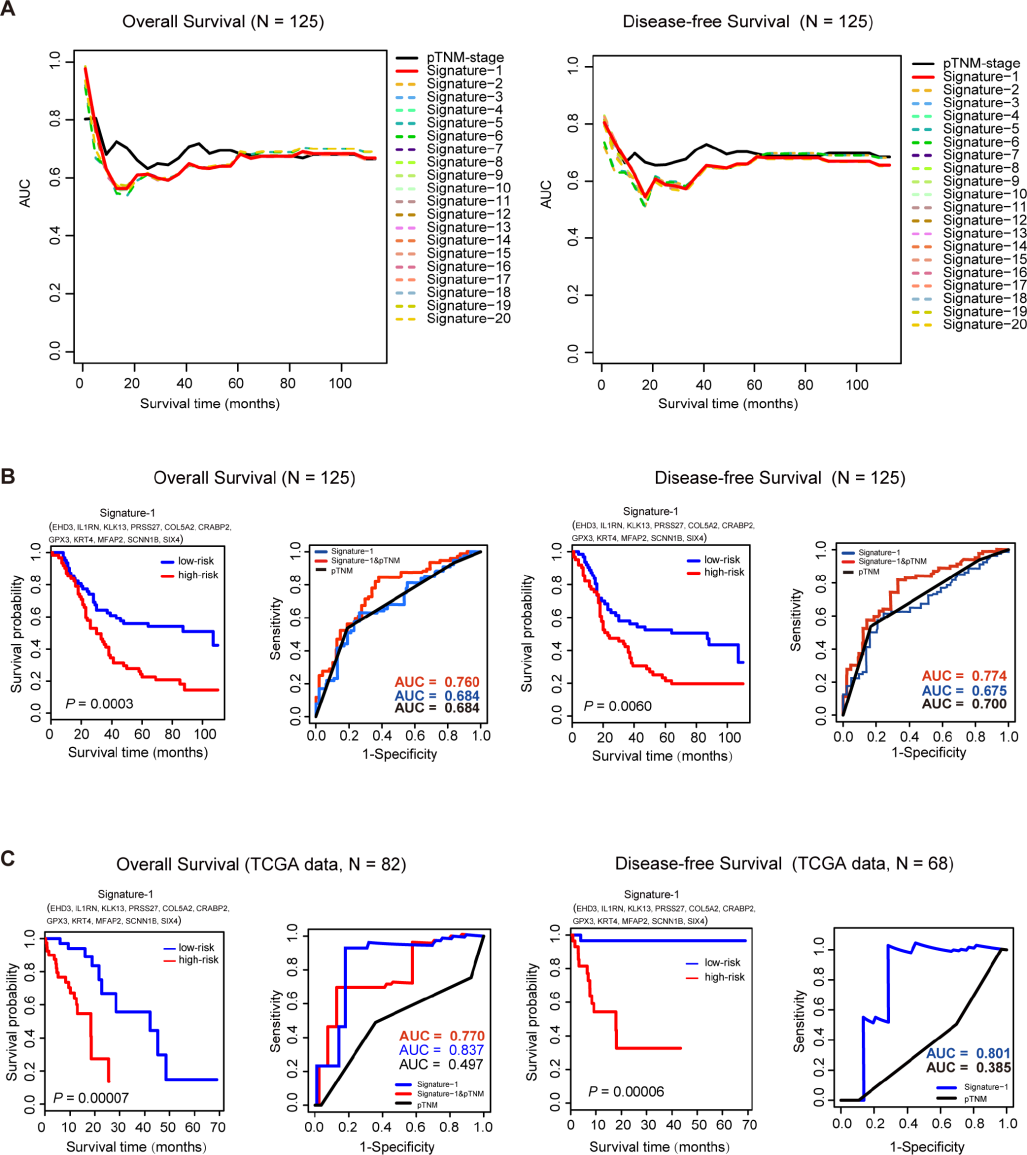
**Survival analysis of 125 ESCC samples and TCGA data sets.** (**A**), Time-dependent AUC curves of overall survival (OS) and disease-free survival (DFS) for the top 20 signatures in 125 ESCC samples. (**B**) For the 125 ESCC samples, Kaplan-Meier curves for overall survival (OS) and disease-free survival (DFS) for Signature-1. ROC analysis shows a better prognostic efficiency of Signature-1 combined with pTNM-stage compared with Signature-1 or pTNM-stage. (**C**) In the TCGA dataset, Kaplan-Meier curves of overall survival (OS) and disease-free survival (DFS)for Signature-1. ROC analysis shows better prognostic efficiency of Signature-1 compared with Signature-1 combined with pTNM-stage or pTNM-stage alone.

In order to explore the clinical significance of the key genes, an Affymetrix IVT microarray of 125 ESCC patients (GSE121931) was used to find the potential prognostic factors (VSIG10L was not scored because it was not detected by the chip). The clinicopathological characteristics of the 125 ESCC patients are shown in Supplementary Table 3. Then survival analysis was used to evaluate the impact of these key genes on the overall survival (OS) and disease-free survival (DFS) of the 125 ESCC patients. First, we conducted 16383 $\left({\text{C}}_{14}^{1}+{\text{C}}_{14}^{2}+{\text{C}}_{14}^{3}+\ldots +{\text{C}}_{14}^{14}\right)$ Cox proportional hazard models by permutating and combining the expression of 14 key genes and OS time or DFS time, respectively. Next, we evaluated the efficiency of each survival model and found that 2,923 models in OS and 1,181 models in DFS were survival-associated (*p*-value<0.01). Although pTNM stage is a crucial prognostic indicator of esophageal cancer [21, 22], the analysis lacks auxiliary biomarkers to aid the accuracy of pTNM [23, 24]. We then performed ROC analysis to compare the prognostic efficiency between these signatures and pTNM stage. Figure 4A and 4B shows the top 20 AUCs of these models for OS and DFS. Then, we found a model (Signature-1) composed of 11 key genes (EHD3, IL1RN, KLK13, PRSS27, COL5A2, CRABP2,GPX3, KRT4, MFAP2, SCNN1B and SIX4) with excellent prognostic capability in both the OS and DFS models, and the AUC of the time-dependent ROC curve was similar to pTNM stage (OS: AUC_Signature-1_ = 0.684, AUC_pTNM_ = 0.684, DFS: AUC_Signature-1_ = 0.675, AUC_pTNM_ = 0.700). In order to understand the clinical significance of Signature-1 better, we associated a series of clinicopathological parameters with this signature in 125 ESCC patients. There was no relevance between Signature-1 and pTNM stage, age and gender (Table 1 and Supplementary Table 4). We found that Signature-1 and pTNM stage were independent of these clinical features and also were independent prognostic factors in OS and DFS (univariate analysis *p*-value<0.05). So we combined Signature-1 with pTNM stage to construct a new model with better prognostic performance (OS: AUC= 0.760, DFS: AUC= 0.774) in our cohort.

**Table 1:** Univariate and multivariate analysis of factors associated with overall survival and disease-free survival

**Variables**	**Univariate analysis**				**Multivariate analysis**			
	*P*-value*	**HR**	**95% CI for HR**		*P*-value*	**HR**	**95% CI for HR**	
			**Lower**	**Upper**			**Lower**	**Upper**
**Overall survival (N = 125)**								
Age (>59 *vs.* ≤ 59)	0.079	1.524	0.953	2.438				
Gender (Female *vs*. Male)	0.253	0.708	0.391	1.280				
pTNM-stage (III *vs*. I+II)	0.000	2.554	1.611	4.048	0.000	2.526	1.621	3.939
Signature-1 (High score *vs*. Low score)^a^	0.016	1.814	1.120	2.938	0.001	2.119	1.342	3.345
**Disease-free survival (N = 125)**								
Age (>59 *vs.* ≤ 59)	0.447	1.195	0.755	1.890				
Gender (Female *vs*. Male)	0.687	0.884	0.485	1.611				
pTNM-stage (III *vs*. I+II)	0.000	2.570	1.620	4.077	0.000	2.582	1.645	4.052
Signature-1 (High score *vs*. Low score)^a^	0.017	1.777	1.110	2.847	0.008	1.856	1.173	2.936
**Overall survival (TCGA data, N = 82)**								
Age (>57 *vs.* ≤ 57)	0.417	1.384	0.631	3.035				
Gender (Female *vs*. Male)	0.168	4.397	0.535	36.165				
pTNM-stage (IV+III *vs*. I+II)	0.019	2.687	1.177	6.138	0.020	2.656	1.167	6.042
Signature-1 (High score *vs*. Low score)^b^	0.000	6.148	2.220	17.023	0.000	6.147	2.239	16.878
**Disease-free survival (TCGA data, N = 68)**								
Age (>57 *vs*. ≤ 57)	0.839	1.117	0.384	3.252				
Gender (Female *vs*. Male)	0.244	29.251	0.100	8527.816				
pTNM-stage (IV+III *vs*. I+II)	0.377	0.558	0.153	2.032				
Signature-1 (High score *vs*. Low score)^b^	0.004	19.462	2.538	149.258	0.005	19.018	2.422	149.333

*Multivariate analysis, Cox proportional hazards regression model. Variables were adopted for their prognostic significance by univariate analysis. ^a^ Low, score< -2.221; high, score ≥ -2.221. ^b^ Low, score< -2.066; high, score ≥ -2.066.

To further investigate the prognostic power of this signature, the test ESCC dataset that was downloaded from TCGA was also used to evaluate the Signature-1 prognostic model. There were 82 ESCC patients with OS time and 68 patients with DFS time in the test datasets. Cox regression analysis was performed first and found that Signature-1 was indeed independent of these clinical features, and pTNM stage also was an independent prognostic factor in OS, but not in DFS (Table 1). In the OS group (n=82), the predictive ability of Signature-1 was significantly better than pTNM stage (AUC_Signature-1_=0.837 vs. AUC_pTNM_=0.497) and the combined model also had a better ability than the TNM stage (AUC_Signature-1&pTNM_=0.770, Figure 4C). In the DFS group (n=68), the predictive ability of Signature-1 was significantly better than pTNM stage (AUC_Signature-1_=0.801 vs. AUC_pTNM_=0.385, Figure 4C). These results demonstrate that Signature-1 has important clinical relevance and is a novel prognostic signature with high accuracy.

### Relationship between DNA methylation and histone modification of the key genes in ESCC

**Figure 5:**
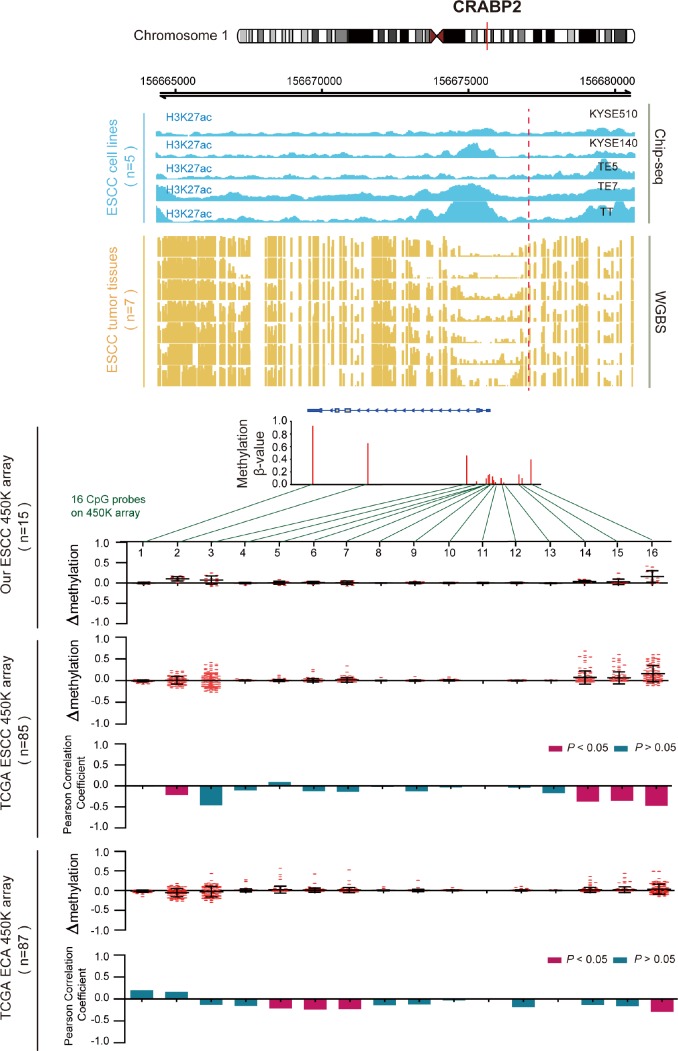
**Inverse trend between DNA methylation and histone modification of CRABP2 in ESCC.** Blue tracks represent the histone modifications in CRABP2 for five ESCC cell lines, and yellow tracks represent its methylation level, as measured by the WGBS assay. All tracks are on the same scale (0-1). Scatter diagrams show the Δβ of CRABP2 in ESCC samples compared with normal samples. Histograms show the correlation between DNA methylation and gene expression of CRABP2.

DNA methylation usually is considered to repress transcription, which is also influenced by histone modifications [25, 26]. In order to further reveal the role of the 11 survival-associated key genes that have aberrant epigenomic regulation in ESCC, we performed H3K27ac ChIP-seq analysis on five ESCC cell lines and whole-genome bisulfite sequencing (WGBS) in seven primary ESCC samples. We define the H3K27ac histone modification level and the WGBS level of a 100bp region which contain the differentially methylated site. Then, we performed the differential analysis between H3K27ac and DNA methylation (WGBS) in this region. After this analysis, we can see that most of these 14 genes have a significant *p*-value between H3K27ac histone modification and methylation (Supplementary Figure 1). Take CRABP2 for example (Figure 5), we examined the methylation β values of all 16 CpG probes that are located on CRABP2 from HM450 array data (Infinium HumanMethylation450) in three cohorts (our data, n=30; TCGA ESCC data, n=85 and TCGA EAC data, n =87). By plotting the change in methylation (Δβ) of ESCC and EAC samples, we identified hypermethylation of promoter CpG loci in ESCC samples, but not in EAC samples. We next calculated the Pearson correlation between each probe’s methylation β value and CRABP2 mRNA expression level in all samples. The uniquely hypermethylated region in ESCC was inversely correlated with CRABP2 expression, but was unchanged in EAC samples. These results indicated that abnormally high methylation of the CRABP2 promoter region was most prominently associated with aberrantly low CRABP2 expression in ESCC. Consistent with the 450K array, the CRABP2 promoter region (TSS±1.5kb) was intensively methylated in seven ESCC samples and corresponded to both a decrease in H3K27acetylation in the same region in ESCC cells and low CRABP2 expression. Interestingly, a previous study indicated that CRABP2 is associated with super-enhancer activity in healthy esophageal tissue [27]. This shows epigenetically modified regions of CRABP2 may affect histone binding and correlates with expression silencing.

**Figure 6:**
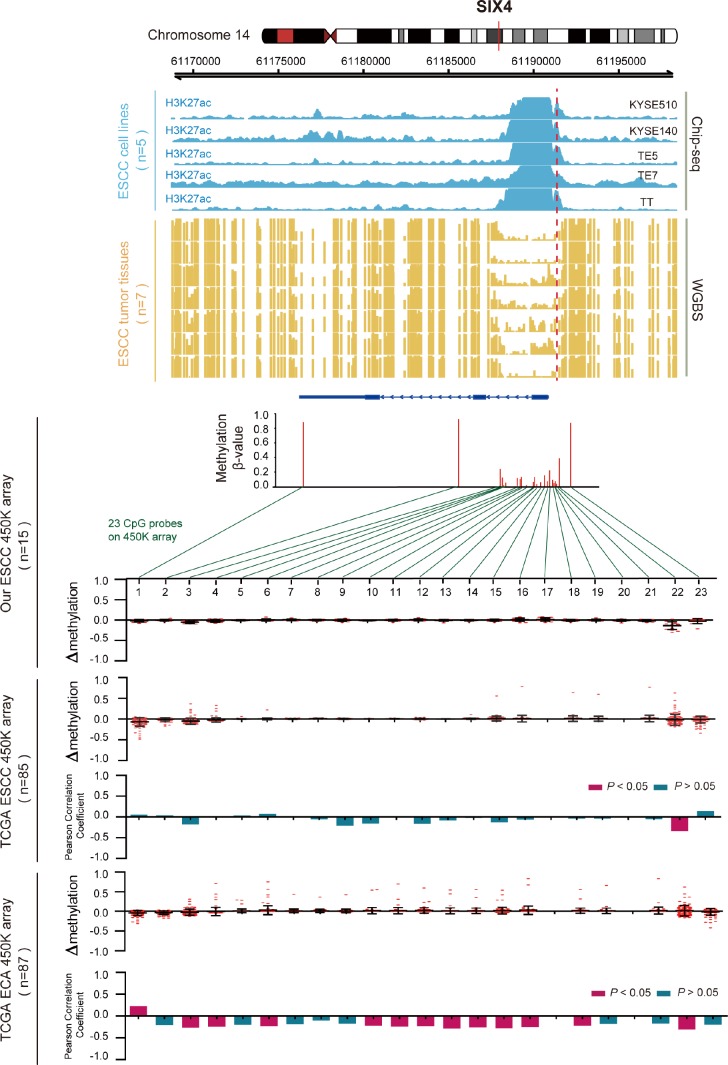
**Inverse trend between DNA methylation and histone modification of SIX4 in ESCC.** Blue tracks represent the histone modifications of SIX4 in five ESCC cell lines, and yellow tracks represent its methylation level, as measured by the WGBS assay, all the tracks are on the same scale (0-1). Scatter diagrams show the Δβ of SIX4 in ESCC samples compared with normal samples. Histograms show the correlation between DNA methylation and gene expression of SIX4.

Regarding the hypomethylated gene SIX4 (Figure 6), we also examined the methylation β values of all 23 CpG probes that were located in SIX4 in the cohorts. We found frequent hypomethylation of the CpG loci at the promoter region in ESCC samples, but not in EAC samples. The uniquely hypomethylated probe in ESCC was inversely correlated with SIX4 expression and was hypermethylated in EAC samples. This indicates that abnormal hypomethylation of the SIX4 promoter was most prominently associated with aberrantly high SIX4 expression in ESCC. ChIP-seq and WGBS data also showed that the SIX4 promoter was weakly methylated in seven ESCC samples, and in the same region, H3K27acetylation was enhanced in ESCC cells and correlated with elevated SIX4 expression (Supplementary Figure 1). Another 12 genes are shown in Supplementary Figure 2. These results further confirmed that aberrant expression of the key genes was regulated by DNA methylation and histone modification.

## DISCUSSION

ESCC is one of the most fatal carcinomas around the world. However, the application of prognostic biomarkers is scarce [28]. In this study, we established a pipeline to find and investigate potentially aberrant genes in ESCC to correlate DNA methylation and mRNA expression by: (1) identifying both differentially-methylated and expressed genes, (2) keeping the genes whose methylation and expression are highly inversely correlated, (3) performing GO and KEGG pathway analysis on the inversely correlated genes, (4) identifying specific key genes by several datasets and verifying their methylation and expression levels by biological experiments, (5) evaluating the prognostic significance of these genes, and (6) exploring the potential molecular mechanisms by examining histone modification and WGBS data.

DNA methylation plays a major role in expression and disease [29–31]. Many researchers have found that super-enhancers may indicate an important role for the key genes in cancers [31, 32]. RNA-Seq also has been applied to identify the functional mRNAs in ESCC [33, 34]. However, single category data may miss some important information related to tumorigenesis and tumor progression. Recently, integrated analysis between DNA methylation and gene expression has been applied to lung adenocarcinoma [35, 36] and cholangiocarcinoma [37]. Aberrant DNA methylation at functional regulatory elements is often accompanied by alterations of histone modifications [38]. So, a comprehensive analysis of DNA methylation and gene expression or histone modification offers a new basis for the diagnosis and treatment of ESCC.

A total of fourteen DMGs were discovered in our integrated analysis, eleven of which (KLK13, PRSS27, COL5A2, KRT4, MFAP2, SCNN1B, SIX4, CRABP2, IL1RN, EHD3 and GPX3) were associated with prognosis of ESCC and also exhibited a negative correlation between DNA methylation and mRNA expression. KLK13 is a member of the tissue kallikrein (KLK) family, and overexpression of KLK13 has been shown to result in an increase in malignant cell behavior [39]. Marapsin (PRSS27) is a trypsin-like serine protease and is highly expressed in normal esophagus [40]. COL5A2, also known as collagen type V alpha 2 chain, has previously been reported to be involved in the pathology of cancer [41, 42], and the protein encoded by KRT4 is a member of the keratin gene family and is related to differentiation [43]. MFAP2 plays an important role in the extracellular deposition [44], and few studies have explored the role of MFAP2 in cancers. SCNN1B encodes the β subunit of the epithelial sodium channel (ENaC), which is essential for the maintenance of body salt and water homeostasis [45]. SIX4 encodes a member of the homeobox family who have been reported to be related with tumorigenesis [46]. Importantly, a model (Signature-1) consisting of key genes highly efficient at predicting both OS and DFS, was used to identify SIX4, suggesting SIX4 could be a potential prognostic biomarker. Four of the key genes (CRABP2, IL1RN, EHD3 and GPX3) are associated with super-enhancers in healthy esophageal tissue and are hypermethylated, with correspondingly low expression, in ESCC samples (Supplementary Table 2). CRABP2 is a crucial component of the RAR pathway and can induce apoptosis in MCF-7 mammary carcinoma cells [47, 48]. IL1RN is an important regulator of the inflammatory response [49]. EHD3 regulates endocytic recycling, along with other EHD family members [50, 51], and it has been considered to be a tumor suppressor in gliomas [52]. GPX3 can catalyze the reduction of peroxides and protect cells against oxidative damage and its hypermethylation has been found in esophageal adenocarcinoma. It also involved in the progression and lymph node metastasis of ESCC [53–55]. These results suggest that the present analysis has identified some potential key genes in ESCC, and their aberrant DNA methylation may affect the function of super-enhancers to lead to abnormal gene expression and influence tumor progression. However, there are still some deficiencies in our research. Although we investigated the aberrant coding RNAs in ESCC, many potential non-coding RNAs regulated by methylation may also play key roles in tumor initiation [56–58].

All in all, this is the first integrated study of epigenomics and transcriptomics to identify key genes underlying the tumorigenic processes of ESCC. Our study reveals many genes with aberrant DNA methylation, which may help to understand the tumor progression of ESCC and to develop novel treatment strategies for patients with ESCC. The eleven genes regulated by methylation are correlated with OS and DFS of ESCC patients and may be potential prognostic biomarkers. In addition, our study combines epigenomics and transcriptomics to provide new insight into the molecular basis of ESCC.

## MATERIALS AND METHODS

### Sample collection and preparation

Information of DNA methylation and gene expression was obtained from 15 patients [34], and clinical information was obtained from 125 patients, all from the Chaoshan District of Guangdong Province, a region of high ESCC prevalence in China. Each sample, comprised of tumor and paired non-tumor tissues, was collected from an individual patient who underwent surgical resection from the Department of Oncological Surgery of the Central Hospital of Shantou City, China. Informed consent was obtained from the participants of this study. This study was approved by the Ethics Committee of the Central Hospital of Shantou City. The 125 ESCC patients’ mRNA expression data used for survival analysis are available publicly at the GEO database under accession number GSE121931.

ChIP-seq data from 5 ESCC cell lines (KYSE510, KYSE140, TE5, TT and TE7) and WGBS data of 7 ESCC tumor tissues were generated previously, by Lin et.al. [59], and visualized in R with bigWig files. The level of H3K27ac histone modification and WGBS methylation was defined by the R package *Gviz*. RNA-Seq data of 85 ESCC patients and 87 EAC patients with matched clinical data were downloaded from TCGA (level 3 data). Five whole genome gene expression data of ESCC and five whole genome gene expression data of other cancers (pancreatic, gastric, colorectal, lung and esophagus adenous cancer) were downloaded from the GEO database. The 10 datasets were selected by the following criteria. First, we focused on gastrointestinal carcinomas, so we selected 4 datasets, including pancreatic cancer, gastric cancer, colorectal cancer and esophagus adenous cancer. Then, we selected a lung cancer dataset because the lung is adjacent to the esophagus. In addition, we also selected 5 ESCC datasets to test whether the results of our data were stable. Finally, all of these datasets should contain paired tumor and normal samples. The GEO accession numbers of the five ESCC datasets were GSE53622, GSE53624, GSE20347, GSE17351, and GSE23400, and the GEO accession numbers of the other cancers were GSE15471, GSE19826, GSE32323, GSE27262 and GSE1420. The Robust Multichip Average (RMA) algorithm was used for processing and normalizing the raw gene expression data.

### DNA methylation microarray

The Illumina Human Methylation450K Array was used to analyze the methylation status of 15 paired ESCC samples. This bead chip covers more than 480,000 methylation sites per sample. The probes were distributed across the promoter, first exon (1^st^ exon), 5′UTR, gene body, and 3′UTR regions [60]. DNA methylation data are available publicly at the GEO database under accession number GSE121930. The raw data were processed by the following methods: 1) probes with a null value were removed, 2) probes located in sex chromosomes were deleted, 3) probes that mapped to multiple genes or were not mapped to genes were removed, and 4) probes containing SNPs were excluded [61].

The Bioconductor R package *minfi* was used for quality control and normalization of the raw data. Probes with a *p*-value < 0.05 were considered differentially methylated. Then the linear regression between DNA methylation β value and gene expression RPKM value for each region of a gene (TSS1500, TSS200, 5’UTR, 1^st^ exon, gene body and 3’UTR) was used to assess which probes were most predictive of the gene expression state [20].

### Identification of differentially-expressed mRNAs

Fifteen ESCC sample expression profiles (SRP064894) were downloaded from NCBI [34]. We first used the “estimate” R package [62] to measure the tissues’ purity of 15 ESCC patients and found that the purity of these 15 patients is more than 50% and the mean purity is 70%. We extracted this data by TopHat and identified the differentially-expressed mRNAs based on the count number expression profile using DESeq (Heidelberg, Germany). In this study, mRNAs with an absolute log2 fold change > 2, and *p*-value < 0.05 are considered differentially-expressed.

### Integration of DNA methylation with gene expression

We integrated the 450K array and RNA-seq data by following analytical process: (1) identification of differentially-methylated and -expressed genes in 15 paired ESCC samples. The differentially-methylated genes were selected by a *p*-value < 0.05, and (2) testing of both differentially-methylated and differentially-expressed genes for a strong association. Pearson correlation analysis was used to identify their correlations. A negative correlation was considered significant for a Pearson correlation coefficient (PCC) < -0.5 and *p*-value < 0.05.

The DAVID Bioinformatics Tool (version 6.8) [63] was used to infer the potential biological processes of methylation-associated genes. Results with *p*-value<0.05 were considered as significant functional categories.

### Cell culture and cell lines

Eight cell lines (KYSE140, KYSE150, KYSE180, KYSE450, KYSE510, TE3 and SHEEC) were used in this study [64]. All cell lines were incubated at 37°C in a humidified atmosphere containing 5% CO_2_.

### qRT-PCR

Total RNA from ESCC tissue or ESCC cell lines was isolated with TRIzol (Invitrogen) as per the manufacturer’s instructions, and the concentration determined with a Nanodrop (Agilent). One microgram total RNA was reverse transcribed into cDNA by a Reverse Transcription System (Promega) according to the manufacturer’s protocol [65]. Quantitative real-time PCR (qRT-PCR) was performed using GoTaq® qPCR Master Mix (TaKaRa) in a 7500 Real-Time PCR System (Applied Biosystems). Primer pairs for target genes used in the PCR assay are listed in Supplementary Table 5.

### Colony formation assay

Cell plate colony formation assays were performed as described previously [65, 66]. Briefly, four cell lines (KYSE140, KYSE150, KYSE180 and TE3) were treated with 100 μM 5-aza-dC for 36 h before being trypsinized and plated into 6-well plates at a concentration of 2,000, 5,000 and 10,000 cells/well. The same four cell lines without 5-aza-dC treatment were used for the control. After washing with PBS, cultures were fixed with 4°C pre-cooled methanol for 15 min, and stained with hematoxylin for 15 min. Colonies were photographed and their sizes calculated with a FluorChem 8900 image analysis system (Alpha Innotech). Each experiment was performed in triplicate.

### Statistical analysis

SPSS 22.0 software (SPSS, Chicago, IL) and R (https://www.r-project.org) were used to perform the statistical analyses. Two-tailed independent sample t tests were used to determine whether differences were significant. Differences were considered statistically significant if the *p*-value < 0.05.

Time-dependent ROC curves were used to assess the prognostic efficiency of the key gene signatures. Kaplan–Meier survival analyses were used to test the survival distributions for ESCC samples. The chi-square test was performed to analyze the association with the clinical signatures, and multivariable Cox regression analysis was used to test whether the signature was independent of other clinical features. R was used to perform all of these analyses. Packages, including *pROC*, *survival* and *survivalROC*, were downloaded from Bioconductor.

## Supplementary Material

Supplementary Figures

Supplementary Table 1

Supplementary Tables 2-5
