# Supplementary material for: Identification of key genes by integrating DNA methylation and next-generation transcriptome sequencing for esophageal squamous cell carcinoma
Source: Aging (Albany NY). 2020 Jan 21;12(2):1332–65. doi: 10.18632/aging.102686 (PMC7053602; doi:10.18632/aging.102686)
Supplement: Supplementary Figures [file aging-12-102686-s002..pdf]

SUPPLEMENTARY FIGURES

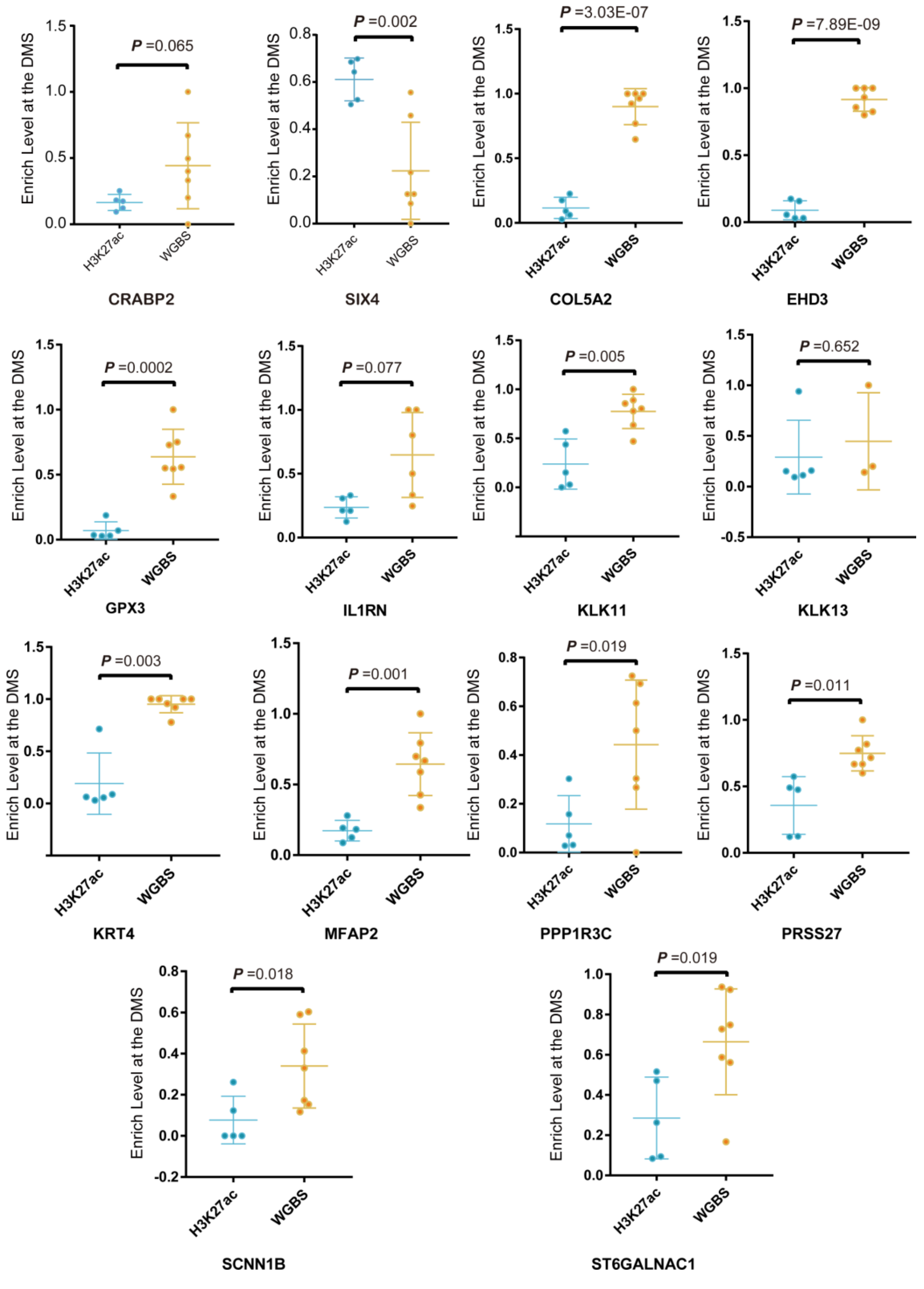

Supplementary Figure 1. Differential analysis between H3K27ac histone modification and WGBS in the differentially methylated site (DMS) of 14 key genes. We detected the enrich level of a 100bp region which contain the DMS. Scatter plot showed the p-value of the differential analysis. Blue spots represent H3K27ac enrich level in 5 ESCC cell lines, whereas the yellow spots represent WGBS level in 7 ESCC samples. T-test were used to determine whether differences were significant. The statistically significant were considered with *p*-value < 0.05.

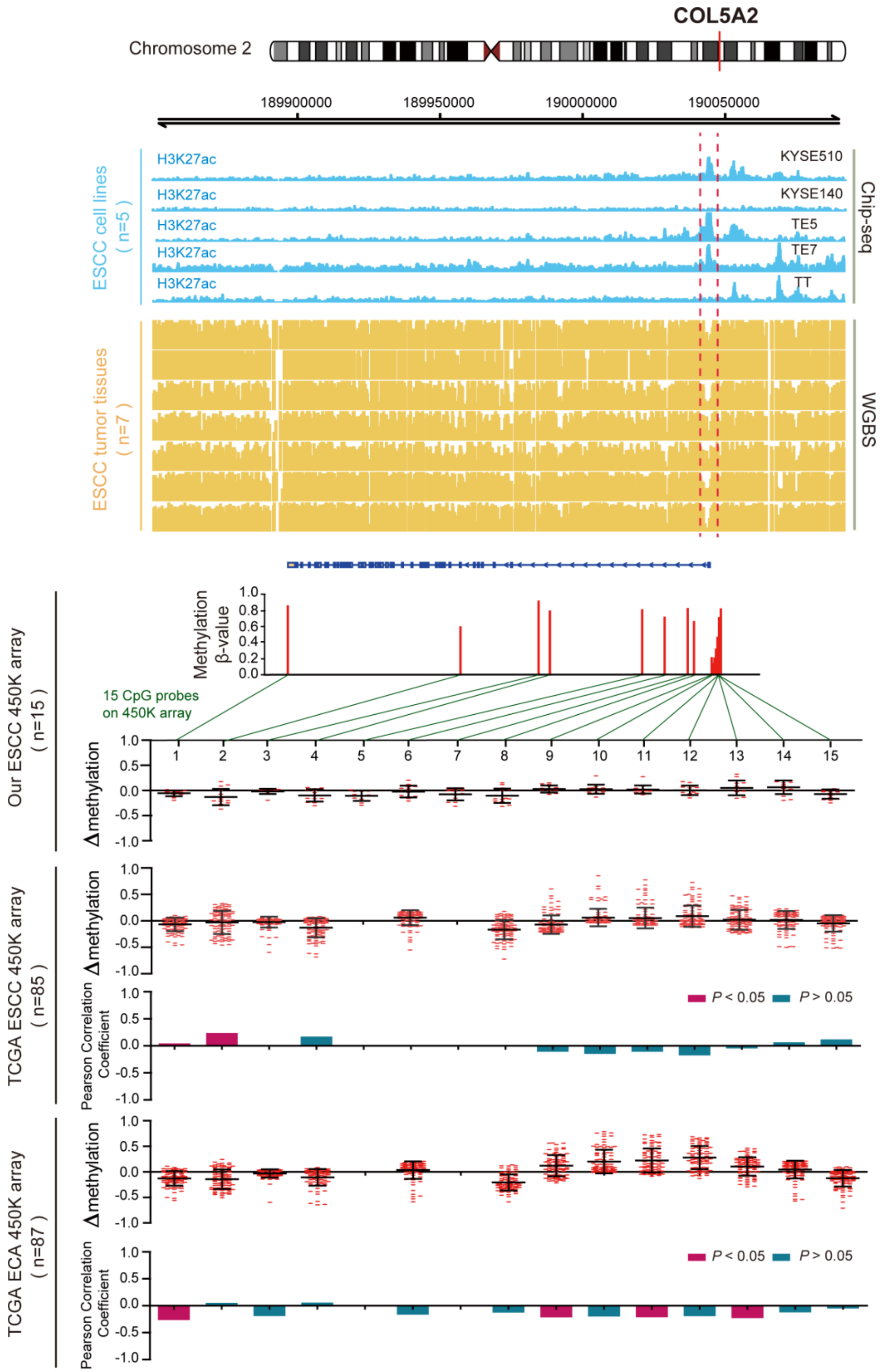
COL5A2
Chromosome 2
189900000
189950000
190000000
190050000
H3K27ac
KYSE510
KYSE140
TE5
TE7
TT
ESCC cell lines
(n=5)
Chip-seq
ESCC tumor tissues
(n=7)
WGBS
Methylation β-value
15 CpG probes on 450K array
Our ESCC 450K array
(n=15)
Δmethylation
TCGA ESCC 450K array
(n=85)
Pearson Correlation Coefficient
P < 0.05
P > 0.05
TCGA ECA 450K array
(n=87)

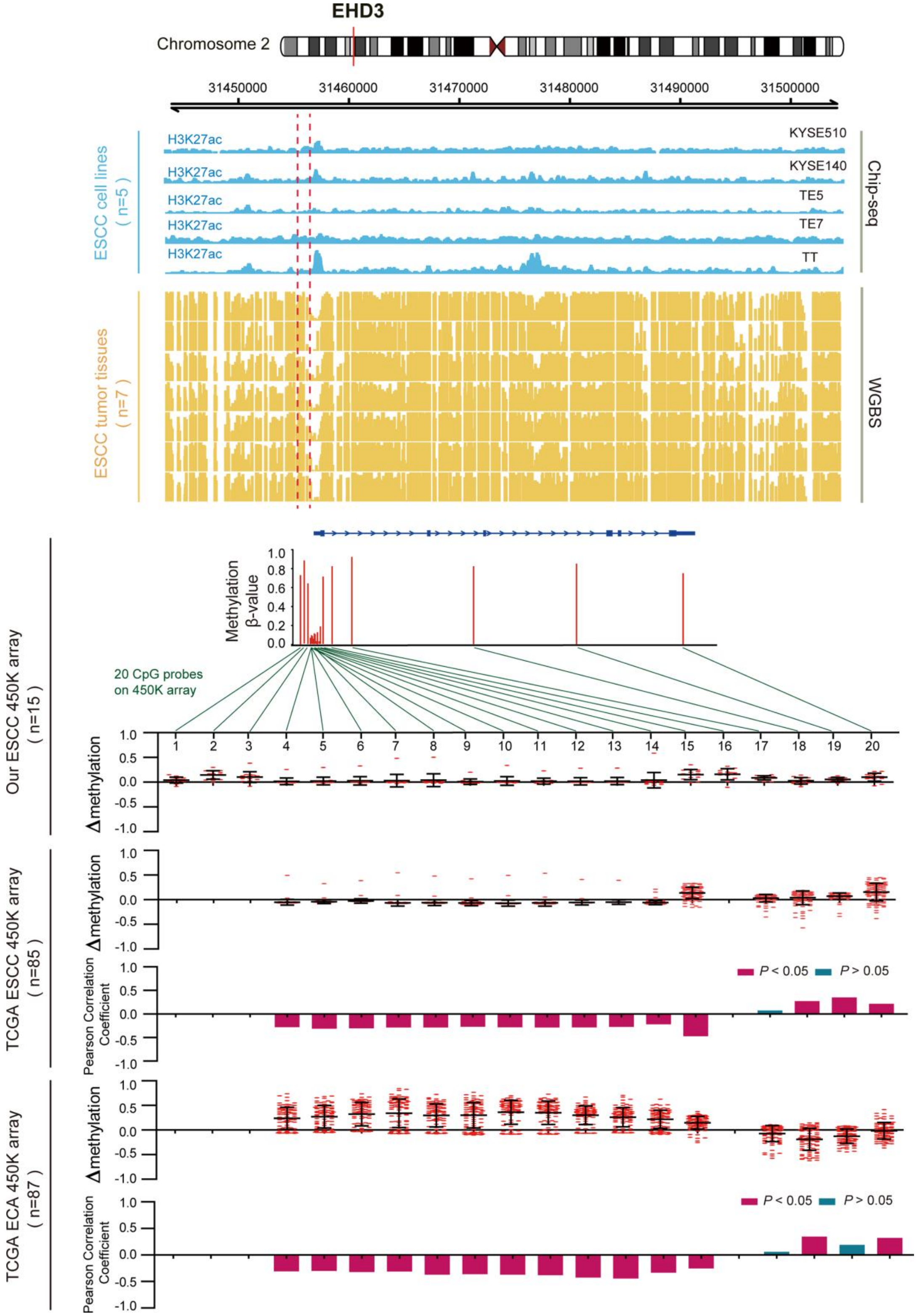

EHD3
Chromosome 2
31450000
31460000
31470000
31480000
31490000
31500000
H3K27ac
KYSE510
KYSE140
TE5
TE7
TT
ESCC cell lines (n=5)
Chip-seq
ESCC tumor tissues (n=7)
WGBS
Methylation β-value
20 CpG probes on 450K array
Our ESCC 450K array (n=15)
Δmethylation
TCGA ESCC 450K array (n=85)
Pearson Correlation Coefficient
P < 0.05
P > 0.05
TCGA ECA 450K array (n=87)

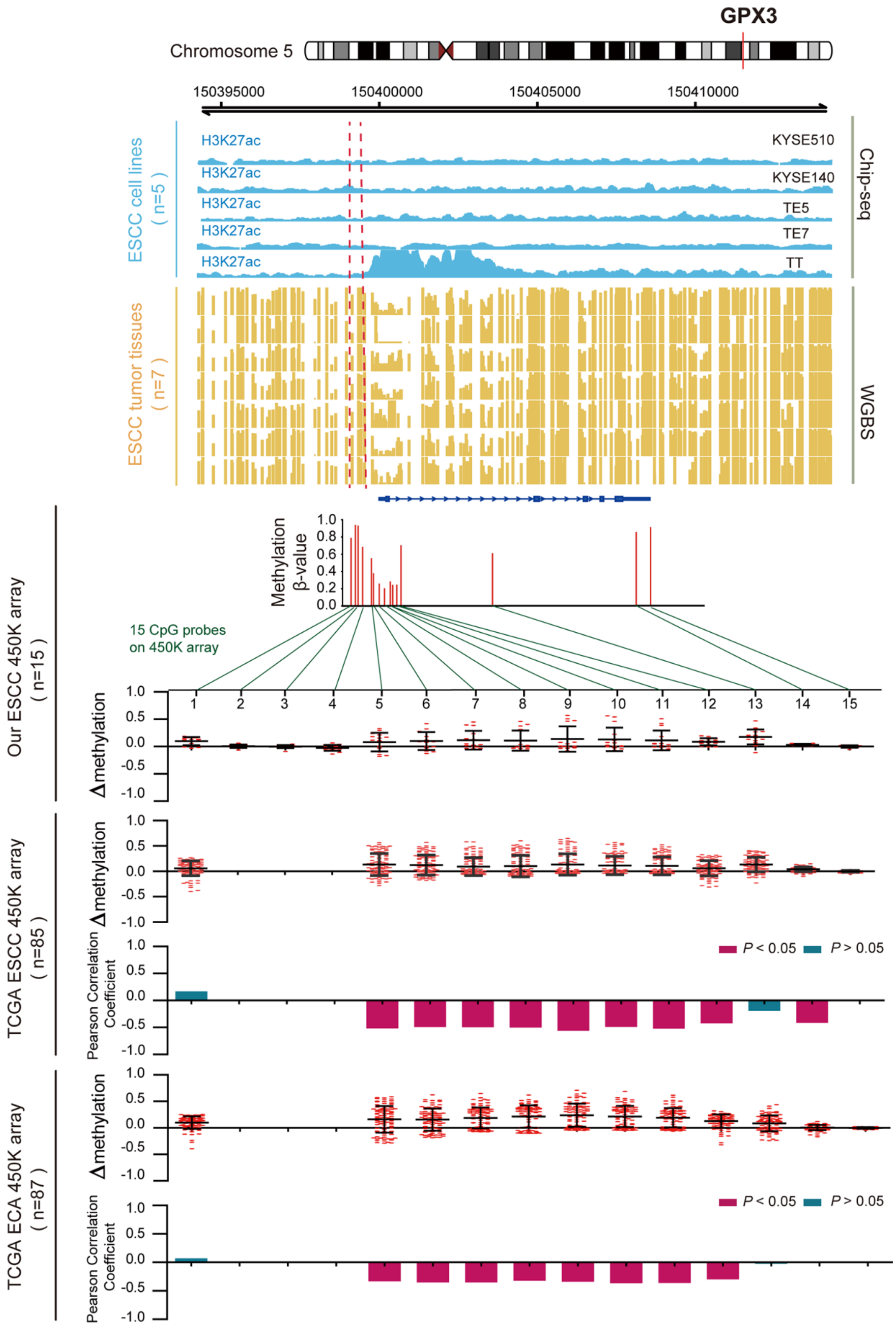

GPX3
Chromosome 5
150395000
150400000
150405000
150410000
ESCC cell lines (n=5)
H3K27ac
KYSE510
KYSE140
TE5
TE7
TT
Chip-seq
ESCC tumor tissues (n=7)
WGBS
Methylation β-value
15 CpG probes on 450K array
Our ESCC 450K array (n=15)
TCGA ESCC 450K array (n=85)
TCGA ECA 450K array (n=87)
Δmethylation
Pearson Correlation Coefficient
P < 0.05
P > 0.05

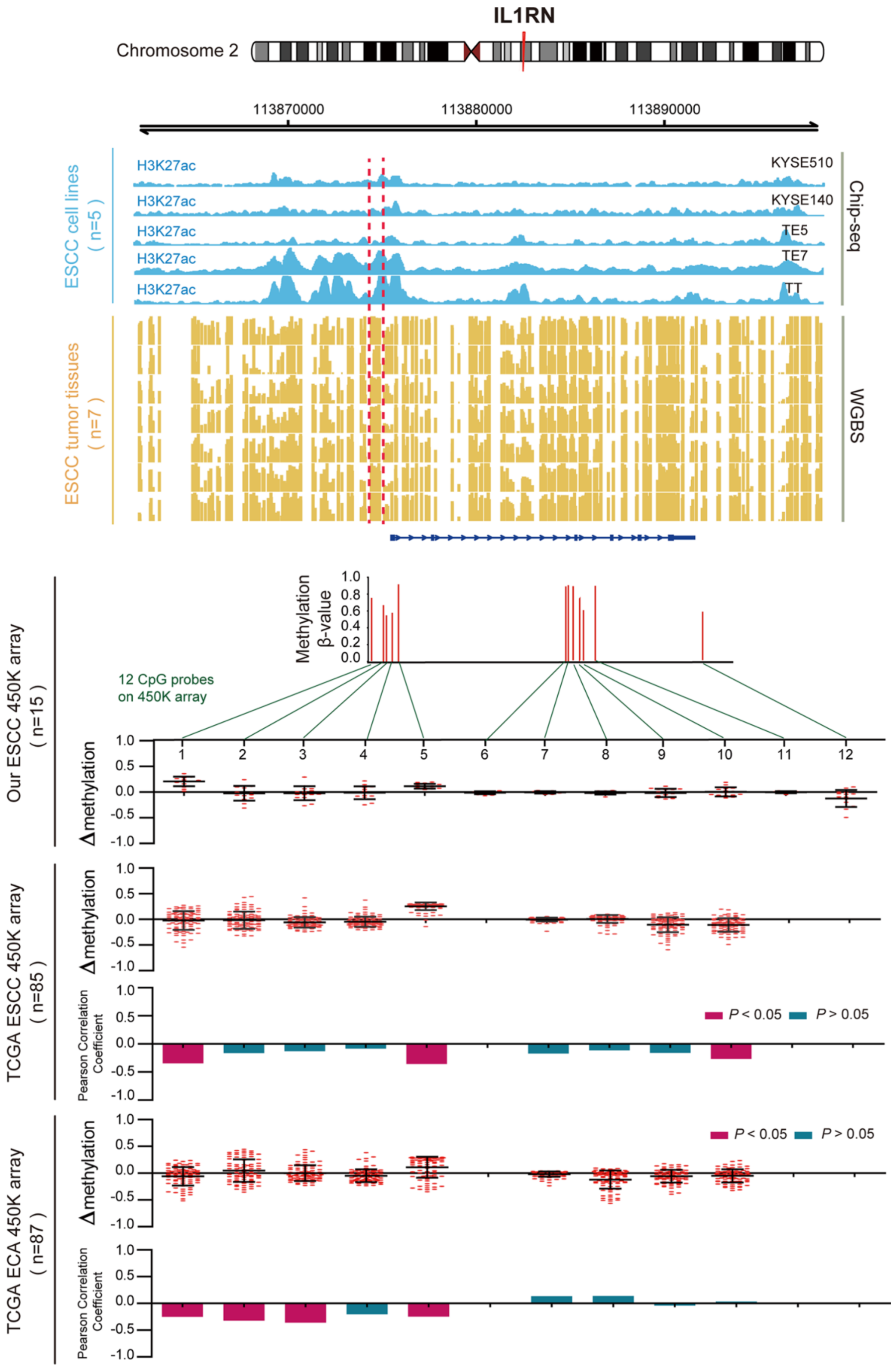

IL1RN
Chromosome 2
113870000
113880000
113890000
ESCC cell lines
(n=5)
H3K27ac
KYSE510
KYSE140
TE5
TE7
TT
Chip-seq
ESCC tumor tissues
(n=7)
WGBS
Methylation
β-value
12 CpG probes
on 450K array
Our ESCC 450K array
(n=15)
Δmethylation
TCGA ESCC 450K array
(n=85)
Pearson Correlation
Coefficient
P < 0.05
P > 0.05
TCGA ECA 450K array
(n=87)

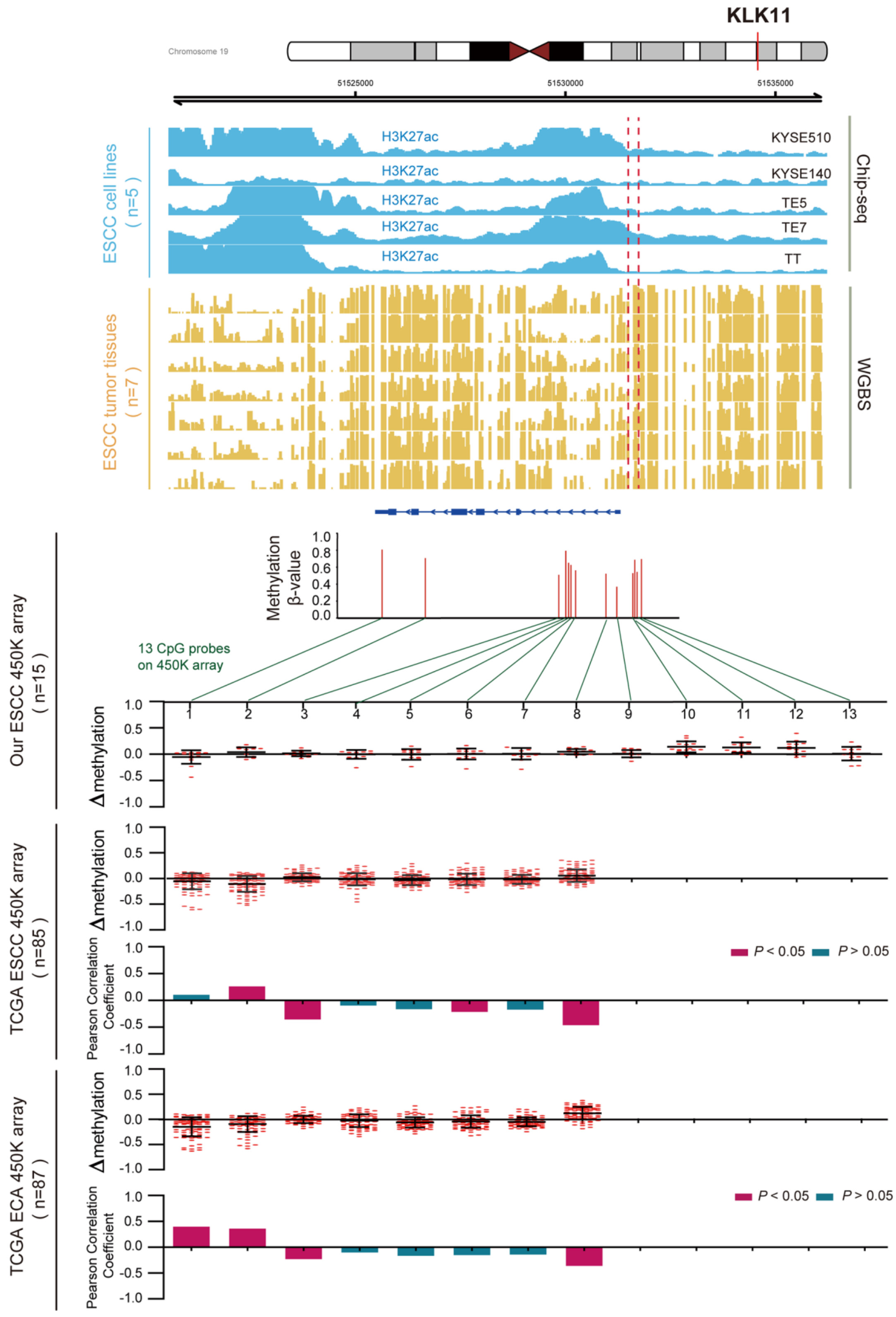
KLK11
Chromosome 19
51525000
51530000
51535000
ESCC cell lines
(n=5)
H3K27ac
KYSE510
KYSE140
TE5
TE7
TT
Chip-seq
ESCC tumor tissues
(n=7)
WGBS
Methylation
β-value
13 CpG probes
on 450K array
Our ESCC 450K array
(n=15)
Δmethylation
TCGA ESCC 450K array
(n=85)
Pearson Correlation
Coefficient
P < 0.05
P > 0.05
TCGA ECA 450K array
(n=87)

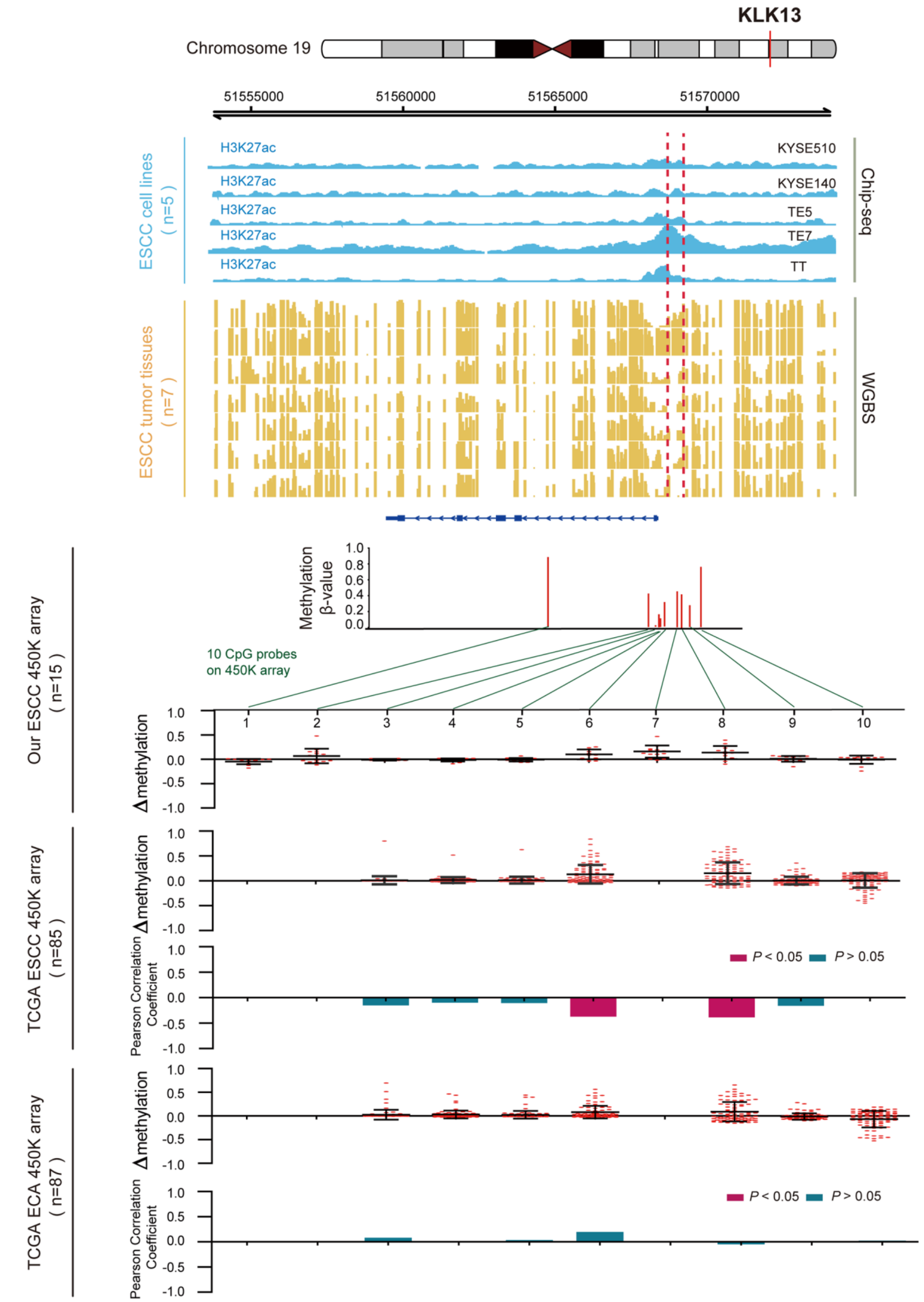
KLK13
Chromosome 19
51555000
51560000
51565000
51570000
ESCC cell lines (n=5)
H3K27ac
KYSE510
KYSE140
TE5
TE7
TT
Chip-seq
ESCC tumor tissues (n=7)
WGBS
Our ESCC 450K array (n=15)
Methylation β-value
10 CpG probes on 450K array
Δmethylation
TCGA ESCC 450K array (n=85)
Pearson Correlation Coefficient
P < 0.05
P > 0.05
TCGA ECA 450K array (n=87)

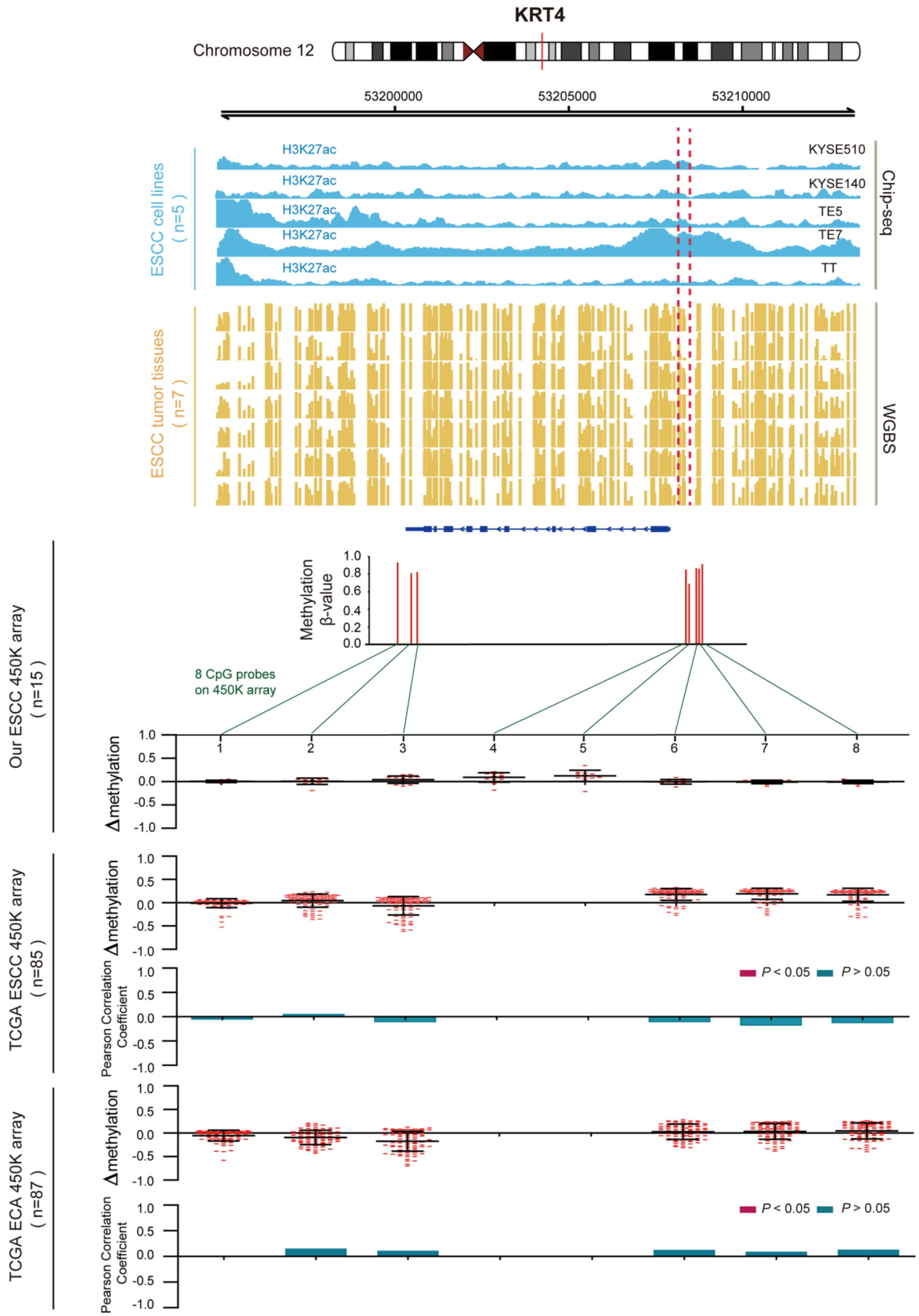
KRT4
Chromosome 12
53200000
53205000
53210000
H3K27ac
KYSE510
KYSE140
TE5
TE7
TT
ESCC cell lines (n=5)
Chip-seq
ESCC tumor tissues (n=7)
WGBS
Methylation β-value
8 CpG probes on 450K array
Our ESCC 450K array (n=15)
Δmethylation
TCGA ESCC 450K array (n=85)
Pearson Correlation Coefficient
P < 0.05
P > 0.05
TCGA ECA 450K array (n=87)

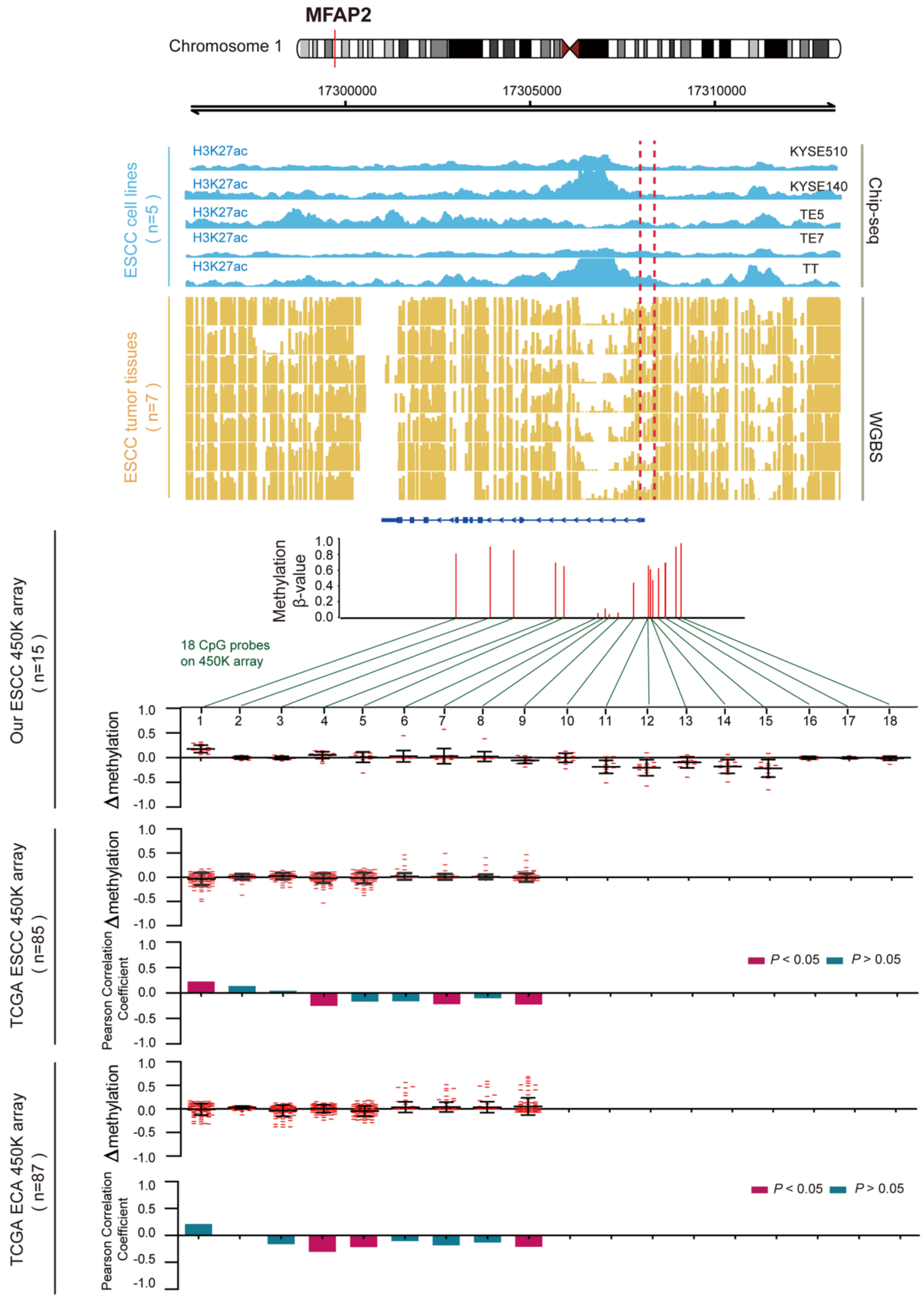
MFAP2
Chromosome 1
17300000
17305000
17310000
H3K27ac
KYSE510
KYSE140
TE5
TE7
TT
ESCC cell lines (n=5)
Chip-seq
ESCC tumor tissues (n=7)
WGBS
Methylation β-value
18 CpG probes on 450K array
Our ESCC 450K array (n=15)
Δmethylation
TCGA ESCC 450K array (n=85)
Pearson Correlation Coefficient
P < 0.05
P > 0.05
TCGA ECA 450K array (n=87)

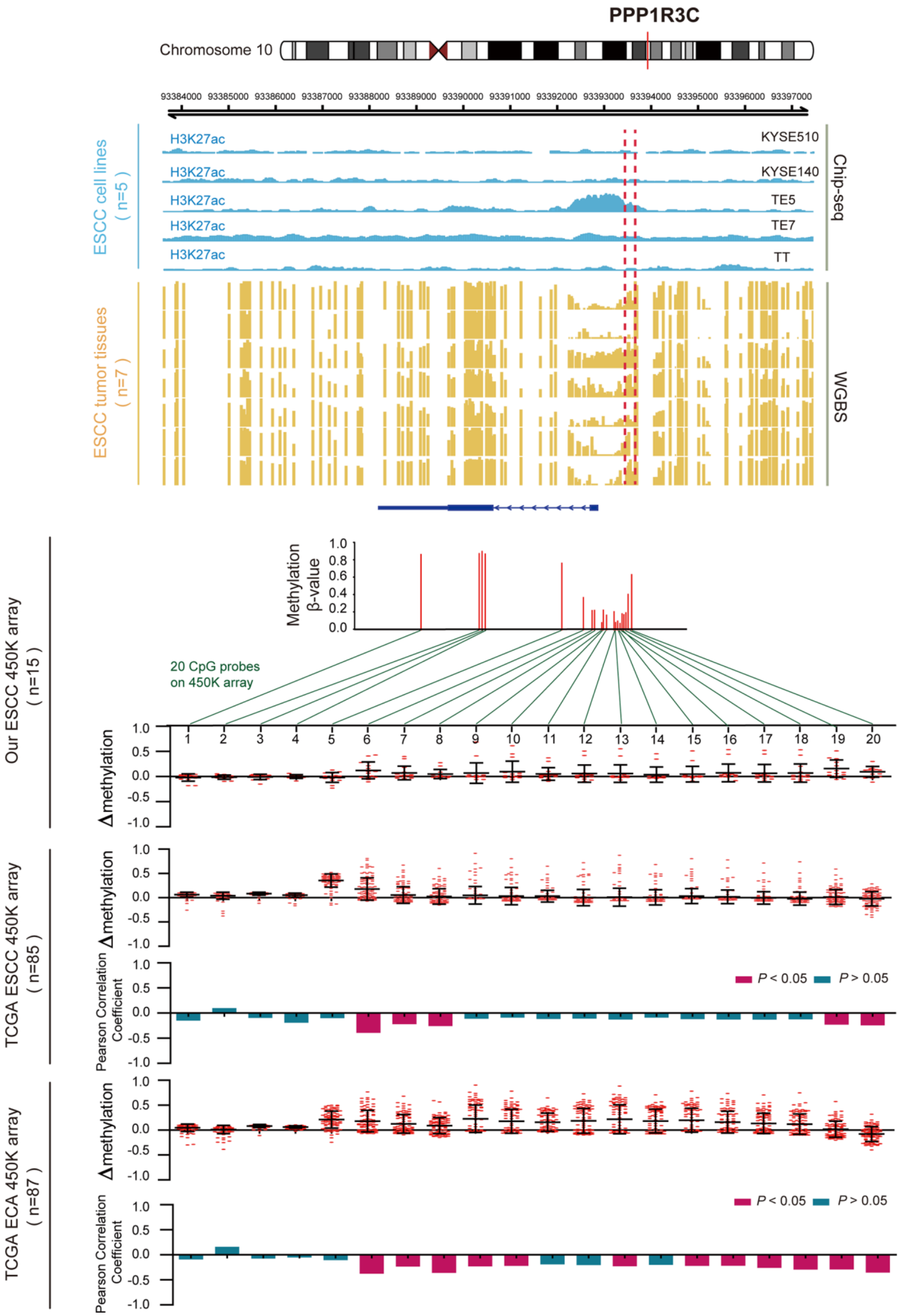

PPP1R3C
Chromosome 10
ESCC cell lines (n=5)
H3K27ac
KYSE510
KYSE140
TE5
TE7
TT
Chip-seq
ESCC tumor tissues (n=7)
WGBS
Our ESCC 450K array (n=15)
Methylation β-value
20 CpG probes on 450K array
Δmethylation
TCGA ESCC 450K array (n=85)
Pearson Correlation Coefficient
P < 0.05
P > 0.05
TCGA ECA 450K array (n=87)

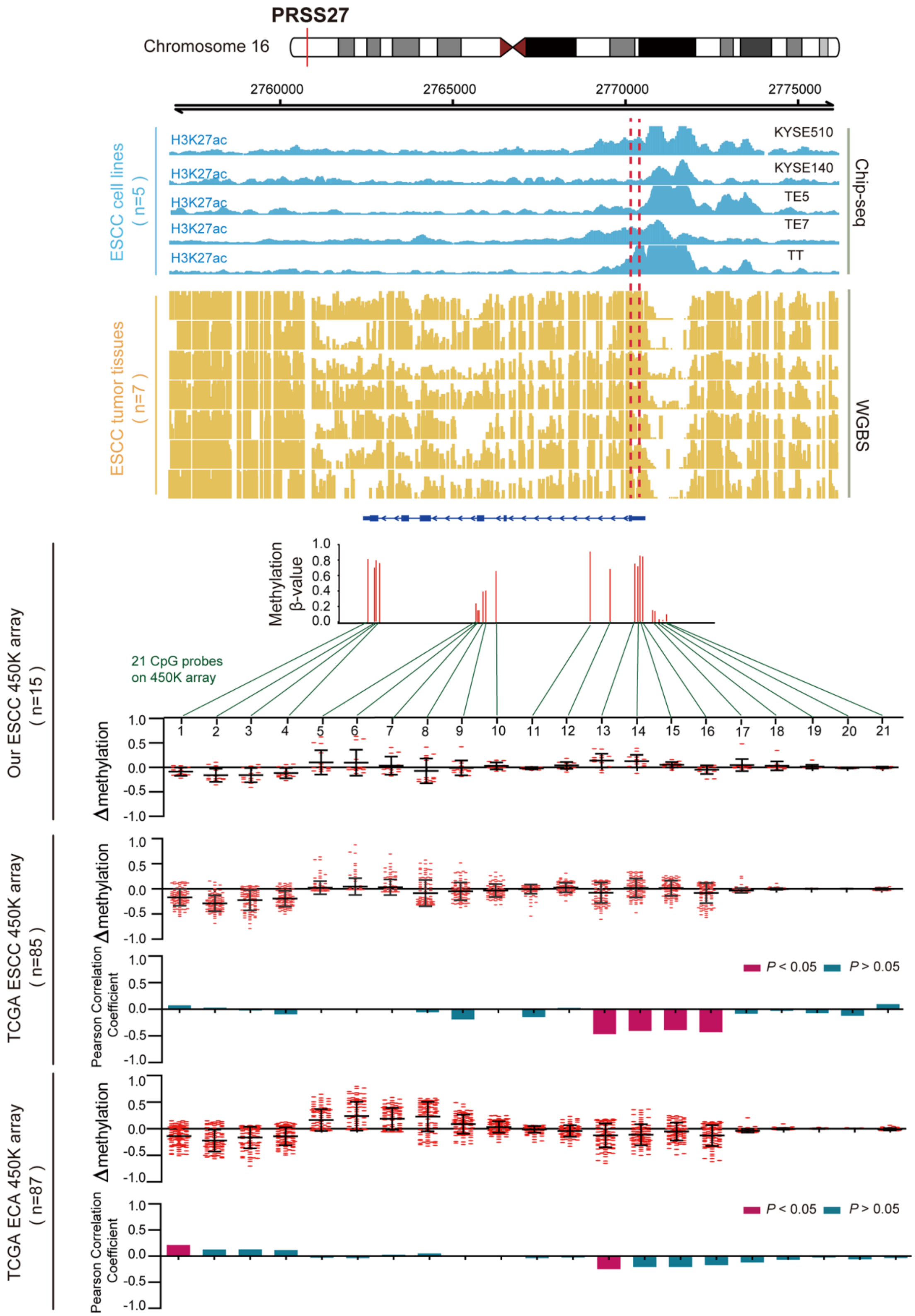
PRSS27
Chromosome 16
2760000
2765000
2770000
2775000
H3K27ac
KYSE510
KYSE140
TE5
TE7
TT
ESCC cell lines (n=5)
Chip-seq
ESCC tumor tissues (n=7)
WGBS
Methylation β-value
21 CpG probes on 450K array
Our ESCC 450K array (n=15)
Δmethylation
TCGA ESCC 450K array (n=85)
Pearson Correlation Coefficient
P < 0.05
P > 0.05
TCGA ECA 450K array (n=87)

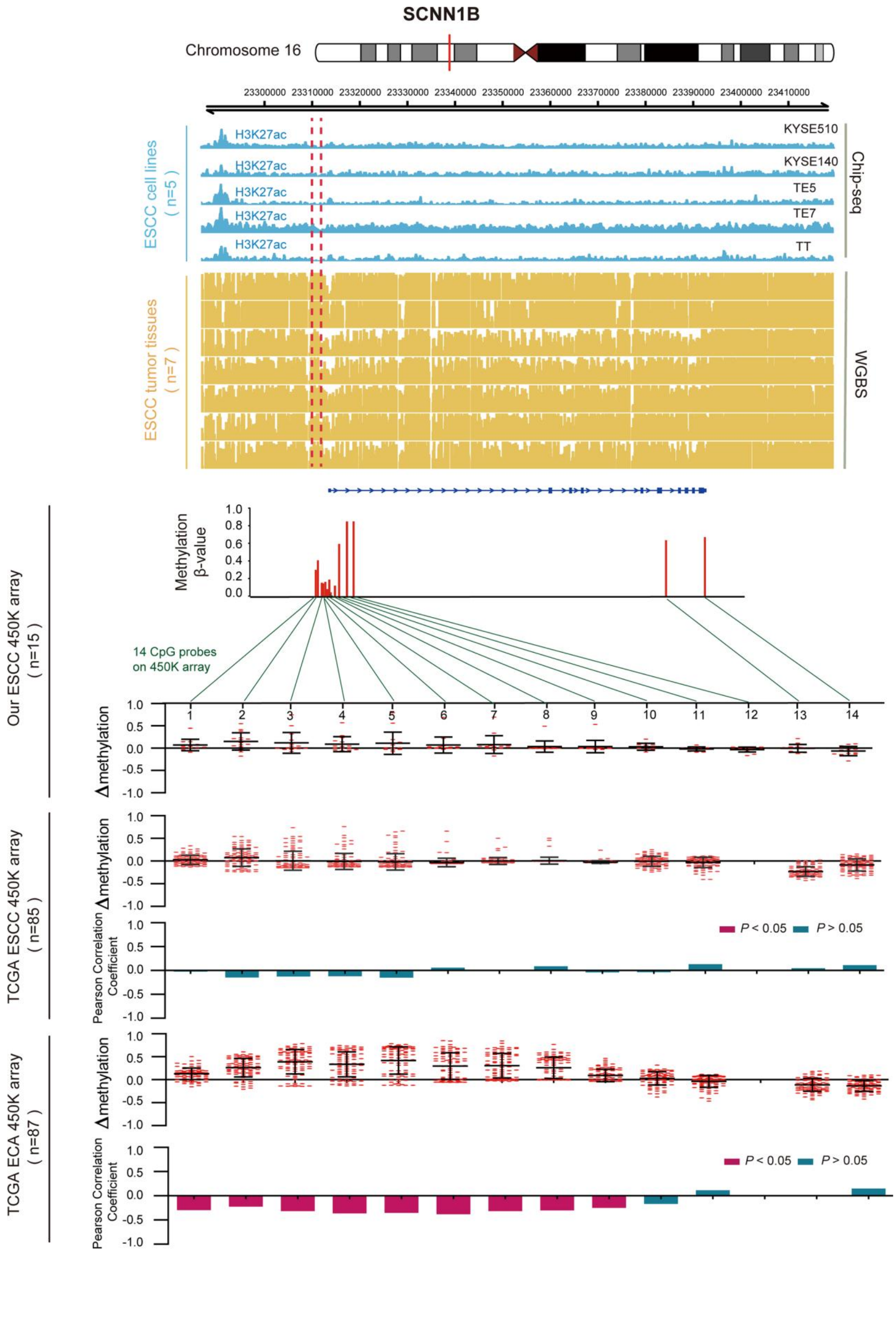

SCNN1B
Chromosome 16
23300000 23310000 23320000 23330000 23340000 23350000 23360000 23370000 23380000 23390000 23400000 23410000
ESCC cell lines (n=5)
H3K27ac
KYSE510
KYSE140
TE5
TE7
TT
Chip-seq
ESCC tumor tissues (n=7)
WGBS
Our ESCC 450K array (n=15)
Methylation β-value
14 CpG probes on 450K array
Δmethylation
TCGA ESCC 450K array (n=85)
Pearson Correlation Coefficient
P < 0.05
P > 0.05
TCGA ECA 450K array (n=87)

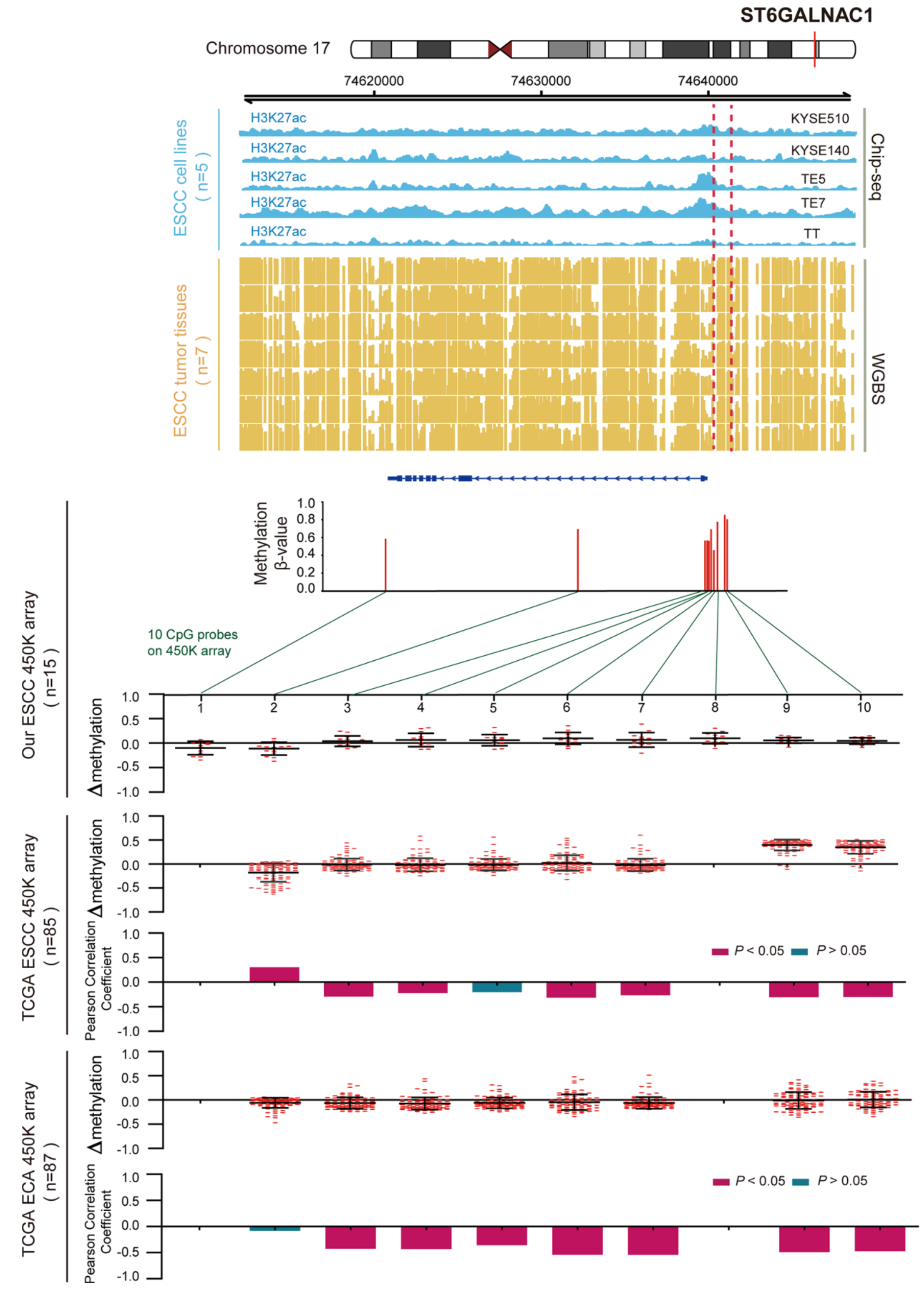

Supplementary Figure 2. Inverse trend between DNA methylation and histone modification of key genes in ESCC cells. Blue tracks represent the histone modifications of these genes in five ESCC cell lines, and yellow tracks represent its methylation level, as measured by the WGBS assay. All tracks are on the same scale (0-1). Scatter diagrams show the Δβ of the genes in ESCC samples compared with normal samples. Histograms show the correlation between DNA methylation and gene expression.
