# Supplementary material for: Identification of key genes by integrating DNA methylation and next-generation transcriptome sequencing for esophageal squamous cell carcinoma
Source: Aging (Albany NY). 2020 Jan 21;12(2):1332–65. doi: 10.18632/aging.102686 (PMC7053602; doi:10.18632/aging.102686)
Supplement: Supplementary Tables 2-5 [file aging-12-102686-s001..pdf]

SUPPLEMENTARY TABLES

Please browse Full Text version to see the data of Supplementary Table 1

Supplementary Table 1. Basic information of 80557 DMPs.

Supplementary Table 2. Basic information of 16 genes.

Gene Symbol	Full Title	Chromosomal Position	Methylation		Expression			Pearson Correlation Coefficient (PCC)	*P*-value of PCC	*SE**
			Status	*P*-value	Level	Log2 (Fold-change)	*P*-value			
CRABP2	Cellular retinoic acid binding protein 2	chr1: 156,667,400-156,677,608	Hypermethylation	2.40E-02	Low	-3.045	6.63E-07	-0.638	1.47E-04	Y
IL1RN	Interleukin 1 receptor antagonist	chr2: 113,873,470-113,893,593	Hypermethylation	3.34E-06	Low	-4.308	1.34E-11	-0.641	1.37E-04	Y
EHD3	EH domain containing 3	chr2: 31,454,880-31,493,260	Hypermethylation	3.48E-02	Low	-2.971	1.77E-06	-0.749	1.96E-06	Y
GPX3	Glutathione peroxidase 3	chr5: 150,397,999-150,408,554	Hypermethylation	2.78E-02	Low	-2.159	7.02E-04	-0.521	3.17E-03	Y
ST6GALNAC1	ST6 N-acetylgalactosaminide alpha-2,6-sialyltransferase 1	chr17: 74,620,839-74,639,900	Hypermethylation	7.03E-03	Low	-2.601	6.33E-03	-0.669	5.38E-05	Y
SCNN1B	Sodium channel epithelial 1 beta subunit	chr16: 23,311,591-23,394,620	Hypermethylation	1.00E-02	Low	-3.830	6.99E-09	-0.544	1.87E-03	N
KLK11	Kallikrein related peptidase 11	chr19: 51,523,487-51,533,290	Hypermethylation	2.90E-04	Low	-3.524	1.21E-07	-0.551	1.59E-03	N
PPP1R3C	Protein phosphatase 1 regulatory subunit 3C	chr10: 93,386,197-93,394,858	Hypermethylation	7.88E-03	Low	-4.137	4.45E-11	-0.684	3.04E-05	N
KRT4	Keratin 4	chr12: 53,198,327-53,209,900	Hypermethylation	4.45E-03	Low	-7.039	1.18E-23	-0.535	2.30E-03	N
SIX4	SIX homeobox 4	chr14: 61,174,256-61,192,852	Hypomethylation	2.00E-02	High	2.904	1.35E-06	-0.562	1.24E-03	N
MFAP2	Microfibril associated protein 2	chr1: 17,298,999-17,310,081	Hypomethylation	3.61E-07	High	3.578	7.51E-03	-0.756	1.17E-06	N
COL5A2	Collagen type V alpha 2 chain	chr2: 189,894,641-190,046,605	Hypomethylation	9.12E-03	High	2.941	2.28E-06	-0.557	1.39E-03	N
KLK13	Kallikrein related peptidase 13	chr19: 51,557,463-51,570,367	Hypermethylation	9.10E-03	Low	-4.901	5.85E-14	-0.596	5.13E-04	N
VSIG10L	V-Set and immunoglobulin domain containing 10 like	chr19: 51,834,784-51,845,375	Hypermethylation	2.07E-02	Low	-3.749	7.09E-10	-0.516	3.54E-03	N
C2orf54	Chromosome 2 open reading frame 54	chr2: 241,825,469-241,835,569	Hypermethylation	8.63E-04	Low	-5.320	2.90E-15	-0.717	8.20E-06	Y
PRSS27	Protease, serine 27	chr16: 2,762,419-2,770,556	Hypermethylation	2.69E-03	Low	-4.543	8.65E-12	-0.616	2.86E-04	N

* SE: super-enhancer around the genes in esophageal tissues or cell lines[27]

Supplementary Table 3. Clinicopathological characteristics of patients with ESCC.

Clinical and pathological indices	Cases	5-year OS (%)	*P*-value [a]	5-year DFS (%)	*P*-value [a]
Specimens	125				
Mean age	59				
Age (years)					
≤59	68	48.5	0.069	44.1	0.156
>59	57	29.8		29.8	
Gender					
Male	102	41.2	0.554	37.3	0.970
Female	23	31.8		39.1	
pTNM stage					
I	13	61.5	0.000	61.5	0.000
II	62	53.2		45.2	
III	50	18.0		22.0	

[a] Log-rank test using the Kaplan Meier method; *P*-value <0.05 was considered significant.
OS, overall survival
DFS, disease-free survival

Supplementary Table 4. Correlation between Signature-1 and clinicopathological characteristics in ESCC (N = 125).

Variables	Signature-1[a]		Chi-square value	*R*	*P*-value
	Low	High			
Age (years)			2.354	0.137	0.125*
≤ 59	38	30			
> 59	24	33			
Gender			2.476	-0.141	0.116*
Male	54	48			
Female	8	15			
pTNM-stage			0.970	-0.041	0.616*
I	7	6			
II	28	34			
III	27	23			

[a]Low, score<-2.221; high, score≥-2.221. * Chi-squared test. *P*-value<0.05 was considered significant.

Supplementary Table 5. qRT-PCR primers used in this study.

Primers	Sequences (5'–3')	Positions
EHD3-qF	CCTTGGAAGAGCATTACCGC	exon 1
EHD3-qR	ACCGCAATGAAGGAGTCTGT	exon 2
KLK11-qF	GAAGACGCGGCTACTCTGTG	exon 3
KLK11-qR	TTGTTGGGGAGGCTGTTGTT	exon 4
KRT4-qF	CTTGGGCAATGACAAAGGGC	exon 2
KRT4-qR	ACCTTGTTCAGGTAGGCAGC	exon 4
ST6GALNAC1-qF	GATATACCGCCCCACCACTG	exon 9
ST6GALNAC1-qR	GTGTAGCCGCTTCCAGACTT	exon 10
COL5A2-qF	AAACTGGGCGGAAGCAAGA	exon 1
COL5A2-qR	GCCATTCTGAGTGCAGGCTA	exon 2
PRSS27-qF	ATTACATCCTCCCCGTGTGC	exon 3
PRSS27-qR	TTGCACTTGGGTGTGTCGAT	exon 4
VSIG10L-qF	CTCAGTCAAGATGGGCGGAA	exon 6
VSIG10L-qR	AGCCTCCACGAGGATATGGA	exon 7
SIX4-qF	TGTCAGTGGCAGCTTCACAA	exon 2
SIX4-qR	GCTCCTTTCCAAGCCTTCCT	exon 3
SCNN1B-qF	CACGAGCAGAGGTCATACCC	exon 6
SCNN1B-qR	CGGGGACCTCAGAACCATTC	exon 7
MFAP2-qF	GTGAGGAACAGTACCCGTGC	exon 7
MFAP2-qR	TAATGACGTACACACGGCGG	exon 8
GPX3-qF	TACGGAGCCCTCACCATTGA	exon 2
GPX3-qR	AGGGAAAGCCCAGAATGACC	exon 3
C2orf54-qF	CAGCCAGTGACCCCACTTAC	exon 4
C2orf54-qR	ATGGCAGAGATCCGGTCCTT	exon 5
IL1RN-qF	TGTGCCTGTCCTGTGTCAAG	exon 4
IL1RN-qR	AAGCGCTTGTCCTGCTTTCT	exon 5
KLK13-qF	TGGCCCTAGTGATCGCCT	exon 1
KLK13-qR	CTGGGAGAAACCCACTGGTC	exon 2
PPP1R3C-qF	TTCGAATTTGTGCAGGCAGC	exon 1
PPP1R3C-qR	TGAATGTGCCAAGCAAAGCC	exon 2
CRABP2-qF	CCGATCGGAAAACTTCGAGGA	exon 2
CRABP2-qR	TTGATCTCCACTGCTGGCTT	exon 3

F: forward primer; R: reverse primer.
